# Stereospecific suppression of active site mutants by methylphosphonate substituted substrates reveals the stereochemical course of site-specific DNA recombination

**DOI:** 10.1093/nar/gkv513

**Published:** 2015-05-20

**Authors:** Paul A. Rowley, Aashiq H. Kachroo, Chien-Hui Ma, Anna D. Maciaszek, Piotr Guga, Makkuni Jayaram

**Affiliations:** 1 Department of Molecular Biosciences, University of Texas at Austin, Austin, TX 78712, USA; 2 Centre of Molecular and Macromolecular Studies, Polish Academy of Sciences, Department of Bioorganic Chemistry, Sienkiewicza 112, 90-363 Lodz, Poland

## Abstract

Tyrosine site-specific recombinases, which promote one class of biologically important phosphoryl transfer reactions in DNA, exemplify active site mechanisms for stabilizing the phosphate transition state. A highly conserved arginine duo (Arg-I; Arg-II) of the recombinase active site plays a crucial role in this function. Cre and Flp recombinase mutants lacking either arginine can be rescued by compensatory charge neutralization of the scissile phosphate via methylphosphonate (MeP) modification. The chemical chirality of MeP, in conjunction with mutant recombinases, reveals the stereochemical contributions of Arg-I and Arg-II. The *S*_P_ preference of the native reaction is specified primarily by Arg-I. MeP reaction supported by Arg-II is nearly bias-free or *R*_P_-biased, depending on the Arg-I substituent. Positional conservation of the arginines does not translate into strict functional conservation. Charge reversal by glutamic acid substitution at Arg-I or Arg-II has opposite effects on Cre and Flp in MeP reactions. In Flp, the base immediately 5′ to the scissile MeP strongly influences the choice between the catalytic tyrosine and water as the nucleophile for strand scission, thus between productive recombination and futile hydrolysis. The recombinase active site embodies the evolutionary optimization of interactions that not only favor the normal reaction but also proscribe antithetical side reactions.

## INTRODUCTION

Phosphoryl transfer reactions in RNA and DNA abound in living cells, and are fundamental to the duplication, transcription, processing and rearrangement of genetic information. In general, these reactions proceed by acid/base catalysis, via a penta-coordinate phosphate transition state (1,2). A prevalent feature of self-catalyzed or protein-catalyzed phosphoryl transfer in nucleic acids is the role of divalent metal ions in the establishment of the active site and/or the promotion of catalysis (3–5). Most systems appear to follow the classical ‘two-metal ion’ model or variations thereof, in which the metals collaboratively stabilize the transition state by activating the nucleophile, facilitating leaving group departure and coordinating a non-bridging phosphate oxygen atom (6–8). A ‘non-canonical’ two-metal mechanism is utilized by type II and type IA topoisomerases, in which one metal promotes transition state stabilization whereas the other indirectly assists catalysis by anchoring DNA (9).

Phosphoryl transfer may also be accomplished by metal-free mechanisms, as exemplified by members of the serine and tyrosine family site-specific DNA recombinases. These enzymes mediate strand cleavage within DNA partners, followed by strand joining across them, with a covalent protein–DNA adduct as the intermediate in the exchange reaction (10–14). Their active sites utilize positively charged side chains to balance the negative charge on the non-bridging oxygen atoms of the scissile phosphate. The active site insignia of tyrosine recombinases is a catalytic hexad cluster comprised of Arg-I–Glu/Asp–Lys–His–Arg-II–His/Trp, together with the tyrosine nucleophile that mediates strand cleavage. Arg-I is nearly invariant in the tyrosine family, while Arg-II is highly conserved, with lysine occupying this position in a small subset of the members (15). Strand cleavage/joining catalyzed by type IB topoisomerases is also metal-independent, and follows the same chemistry as tyrosine recombination (16). The tyrosine nucleophile in the topoisomerases is assisted by a catalytic cohort remarkably similar to the conserved active site cluster of the recombinases, and includes the Arg-I/Arg-II pair. The present analysis is focused on the mechanistic roles of the arginine duo (Arg-I and Arg-II) of the catalytic hexad, in particular their stereo-specific interactions with the non-bridging oxygen atoms of the scissile phosphate.

**Figure 1.**
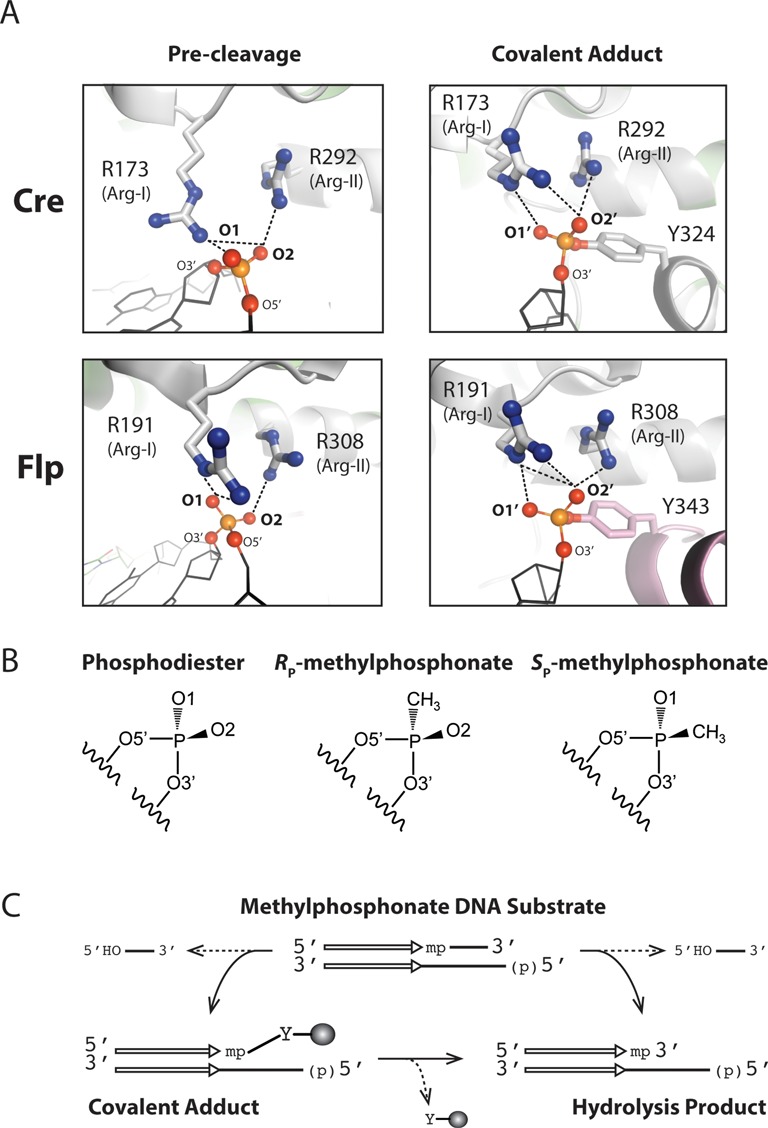
Interactions of the conserved arginine duo of the tyrosine recombinase active site with the scissile phosphate. (**A**) In these representations, the non-bridging oxygen atoms of the scissile phosphate are labeled O1 and O2 in the intact DNA backbone. The corresponding labels are O1’ and O2’ in the cleaved tyrosyl intermediate. Further details of the nomenclature are given in the text. The O1 and O2 nomenclatures are simplified from the current OP1 and OP2 nomenclatures (previously O1P and O2P), respectively, in PDB (protein data bank). In the structures of Cre–DNA and Flp–DNA complexes, contacts made by Arg-I and Arg-II with O1 and O2 are shown in the left panels (34,35) (PDB: 4CRX; 1M6X). Arg-I refers to the more N-terminally located arginine (Arg-173 in Cre; Arg-191 in Flp). These contacts are refashioned in the cleaved tyrosyl intermediate (right panels) (20,34) (PDB: 1CRX; 1M6X). The distances of Arg-I and Arg-II side chain groups from the non-bridging oxygen atoms in the structures shown here are tabulated in Table 1. In the ‘pre-cleavage’ structures for Cre and Flp, the active site tyrosines (Tyr-324 in Cre; Tyr-343 in Flp) are positioned quite differently, and are not shown here. In the structures of the covalent adduct, Tyr-343 of Flp is shaded differently to indicate that, unlike Tyr-324 of Cre, it is donated in *trans* from a neighboring Flp monomer. (**B**) Replacement of the non-bridging O1 atom in the phosphodiester gives *R*_P_-methylphosphonate (*R*_P_-MeP); the corresponding substitution of O2 gives *S*_P_-MeP (see also Supplementary Figure S1). (**C**) Strand cleavage in an MeP-half-site is schematically illustrated. The recombinase binding element is represented by the pair of parallel arrows, and the scissile MeP by ‘mp’. Attack of MeP by the active site tyrosine nucleophile gives a 3′-MeP-tyrosyl adduct, which may be hydrolyzed to the 3′-methylphosphate. The hydrolysis product may be generated in a single step if water out-competes the tyrosine nucleophile.

Cre and Flp, coded for by bacteriophage P1 and the budding yeast plasmid 2 μm circle, respectively, are mechanistically closely related tyrosine recombinases (13,14). Cre is thought to promote faithful segregation of the unit copy phage/plasmid by resolving dimeric genome circles (resulting from homologous recombination) back into monomer circles (17). Flp is required for copy number maintenance of the yeast plasmid by performing an appropriately timed, replication-coupled recombination event that triggers an amplifying mode of DNA replication (18,19). The Arg-I and Arg-II residues in Cre are Arg-173 and Arg-292, respectively; the corresponding residues in Flp are Arg-191 and Arg-308 (Figure 1A). There is an important distinction between Cre and Flp in the assembly of their active sites. The Cre active site is contained entirely within a monomer (20). The Flp active site is assembled by the donation of the tyrosine nucleophile from one monomer to the pro-active site (containing the hexad cluster) of an adjacent monomer (21,22). However, the engagement of the scissile phosphate by the key catalytic residues is quite similar between the ‘self-contained’ and ‘shared’ active sites (Figure 1A).

The substitution of Arg-I or Arg-II in Cre and Flp by charge-neutral residues almost completely abolishes the chemical competence of their respective active sites (15,23,24). However, strand breakage by Arg-I-to-Ala or Arg-II-to-Ala mutants can be restored by electrostatic compensation in the DNA substrates (25–28). In these modified methylphosphonate (MeP) substrates (Figure 1B), one of the non-bridging oxygen atoms of the scissile phosphate is replaced by the methyl group, thereby eliminating the nominal unit negative charge in the ground state. All four Arg-to-Ala point mutants (Cre(R173A) and Cre(R292A); Flp(R191A) and Flp(R308A)) are active on MeP ‘half-site’ substrates, and yield strand scission by one of two mechanisms (Figure 1C). The Cre mutants utilize the active site tyrosine (Tyr-324) nearly exclusively as the cleavage nucleophile (27,28). In the Arg-II-to-Ala Flp mutant reaction, the nucleophile role is taken over almost completely by water (26). In the case of the Arg-I-to-Ala Flp mutant, the catalytic tyrosine (Tyr-343) serves as the principal nucleophile, the contribution from water nucleophile being minor (25).

The MeP modification, in addition to its electrostatic effect, confers chemical chirality on the normally symmetric phosphate center. We have taken advantage of this asymmetry to dissect the stereochemical contributions of Arg-I and Arg-II to recombination. Furthermore, the MeP reactions with Cre and Flp variants lacking Arg-I or Arg-II reveal mechanistic features of the tyrosine recombinase active site that could not have been gleaned from standard reactions utilizing native, phosphate-containing DNA substrates. The outcomes from the present analyses underscore the general utility of methylphosphonate substrates in their racemic and stereochemically pure forms for probing the electrostatic and stereochemical attributes, respectively, of phosphoryl transfer active sites that act on nucleic acids.

## MATERIALS AND METHODS

### Synthetic methylphosphonate (MeP) containing oligonucleotides

The sequences of the oligonucleotides utilized in this study are listed in Supplementary Table S1. Racemic mixtures of MeP-oligonucleotides were purchased from Trilink Technologies (San Diego, CA, USA). Stereochemically pure *R*_P_ and *S*_P_ forms of MeP-oligonucleotides were synthesized according to published protocols (29,30), as outlined in a previous study (26).

### Cre, Flp and their active site mutants

Purification of Cre and Flp recombinases and their mutant derivatives were performed according to previously described procedures (26,27).

### Assembly of half-site substrates

The oligonucleotides for the scissile and non-scissile strands of a half-site substrate were first phosphorylated at their 5′ ends using ^32^P-labeled and unlabeled ATP, respectively, in a T4 polynucleotide kinase reaction. They were purified by electrophoresis in urea-polyacrylamide gels, followed by extraction from the gels. Hybridization of the oligonucleotides was carried out as described previously (25).

### MeP-half-site reactions *in vitro*

Half-site reactions were performed after optimizing conditions, as in earlier studies (25,28). The molar ratio of Flp (or a Flp variant) to the Flp binding site was maintained at ∼8 (∼7 nM half-site; ∼60 nM Flp). The corresponding ratio of Cre (or a Cre variant) to the Cre binding element was ∼10 (∼7 nM half-site; ∼70 nM Cre). At these ratios, substrate binding was saturated. Separate binding assays verified that the binding affinities of the wild type and mutant recombinases to MeP-half-sites were similar (within a factor of 2 to 3). Reactions were stopped by adding SDS to a final concentration of 0.1%, and split into two equal portions. One half was analyzed by 12% SDS-polyacrylamide (acrylamide/bis-acrylamide 29:1) gel electrophoresis to detect the covalent protein–DNA adduct. DNA was purified from the other half by phenol–chloroform extraction and ethanol precipitation before electrophoresis in 12% urea–polyacrylamide (acrylamide/bis-acrylamide 19:1) sequencing gels to detect the hydrolysis product. The phenol–chloroform extraction step traps the covalent adduct containing the denatured protein at the organic-aqueous interphase, separating it from the uncleaved DNA and the hydrolysis product.

### Quantification of reaction products and determination of stereochemical bias

Substrate and product bands, detected by a phosphor storage screen (Bio-Rad), were scanned using a Typhoon Trio Phosphorimager (GE Healthcare). Image analysis and quantification of band intensities were performed using the software Quantity One (version 4.5.1; Bio-Rad) (25). The extent of reaction at a given time point was estimated as a ratio of the product band intensity to the sum of substrate and product band intensities. For determining the stereochemical bias, these values were corrected by subtracting the background from control reactions. The bias favoring the *S*_P_ reaction (*S*_P_/*R*_P_) or the *R*_P_ reaction (*R*_P_/*S*_P_) were calculated as the mean ratio of the corrected values for time points within the early (linear) phase of the reactions. As the product yield by Flp(R191G) was quite low, its bias was derived from a single time point of 48 h. The bias estimates were qualitatively similar to those derived from the pseudo-first order rate constants for the *R*_P_ and *S*_P_ reactions (see below).

### Determination of kinetic constants: endpoints of reactions

The rate constants were determined by assuming the reaction to follow pseudo-first order kinetics. Furthermore, direct MeP hydrolysis and formation of the tyrosyl intermediate first followed by its hydrolysis were both treated as single-step reactions in the kinetic analyses. The Flp reaction would require, at least for the formation of the tyrosyl–DNA adduct, dimerization of a Flp-bound half-site because of the shared active site of Flp (21,22). Association of Flp-bound half-sites to form dimeric and higher-order complexes has been demonstrated (31).

For reactions of Arg-I and Arg-II mutants of Cre, and the Arg-I mutants of Flp (which yield the hydrolysis product primarily via the tyrosyl intermediate), the endpoints gave 50% or less conversion of the preferred MeP-half-site stereoisomer. This result is rather surprising, given the molar excess of a recombinase mutant over the half-site in these reactions. At least part of the reason for the incomplete reaction is the inactivation of the recombinase during prolonged incubation. Additionally, since a recombinase-bound half-site dimer harbors only one functional active site at a time (half-of-the sites activity) (20,22,32), strand cleavage is restricted to one half-site within such a complex. The half-site with a covalently bound recombinase, or the hydrolysis product derived from it, may be less efficient than a native half-site in promoting cleavage within an associated half-site partner. The net product formation may also be limited by the rate of dissociation of a cleaved complex, which would allow an uncleaved (but recombinase-bound) half-site to form productive association with a second such half-site.

The reactions of the Arg-II mutants of Flp may not require dimerization of the half-sites bound by them, as these mutants promote direct hydrolysis without involvement of the active site tyrosine. The product yields for Flp(R308A) and Flp(R308Q) were similar (∼40%), and less than that for Flp(R308K) (∼60%), at the 8 h time point in the preferred *S*_P_-MeP reactions. Flp(R308E) showed rapid kinetics, hydrolyzing ∼90% of the input *S*_P_-MeP substrate in 30 min.

## RESULTS

### MeP-modification: introduction of chirality at the scissile phosphate center

The literature on tyrosine recombinases and type IB topoisomerases contain inconsistencies in the labeling and nomenclature of the scissile phosphate and its modifications. In order to avoid confusion, we first spell out the conventions followed here, as depicted by the labeling scheme in Figure 1A. In the uncleaved DNA backbone, the non-bridging oxygen atoms are labeled as O1 and O2. Following cleavage via ‘in-line’ attack by the tyrosine nucleophile, and formation of the phosphotyrosyl bond, these oxygen atoms are renamed as O1’ and O2’, respectively, to denote the ‘inversion’ of phosphate configuration. When the O1 oxygen is replaced by the methyl group, the resulting MeP has the *R*_P_ configuration; analogous replacement of O2 gives the *S*_P_ form of MeP (Figure 1B and Supplementary Figure S1A and B). These stereochemical assignments are based on the Cahn-Ingold-Prelog priority rules, explained under Supplementary Figure S1. To be consistent with this nomenclature, the O1 and O2 labels in the poxvirus topoisomerase and Cre recombinase structures of their respective vanadate transition state mimics (15,33) need to be reversed. A more recent representation of the Cre structure (13) conforms to the O1 and O2 designations in Figure 1. The interpretations of the experimental analyses to follow can be greatly simplified by keeping in mind that O2 is intact in the *R*_P_ form of MeP, and O1 is intact in the *S*_P_ form.

### MeP-half-site reactions

The MeP-half-site substrates (26,27) harbor one recombinase binding element and one scissile MeP (Figure 1C). Strand breakage in a half-site is virtually irreversible, as the short trinucleotide product carrying a 5′-hydroxyl end would diffuse away from the reaction center. Cleavage mediated by the active site tyrosine would result in a covalent recombinase-DNA adduct. A pseudo-joining reaction, yielding a hairpin product, is prevented by phosphorylating the 5′-hydroxyl on the non-scissile strand. An alternative to the normal cleavage reaction is direct attack of the MeP backbone by water. The hydrolytic product can also be formed in two steps, by the action of water on the MeP-tyrosyl bond of the recombinase-DNA adduct.

### The logic of probing the stereochemical contributions of Arg-I or Arg-II

In the crystal structures, Arg-I in both Cre and Flp is seen to contact O1 when the DNA backbone is uncleaved (Figure 1A, left panels; Table 1) (34,35). Arg-173 in Cre makes a single hydrogen bond with O1, while Arg-191 in Flp makes two bidentate hydrogen bonds with O1. Furthermore, Arg-173 may form a hydrogen bond with O2 as well (3.3 Å; Table 1). Arg-II in Cre and Flp forms a tight hydrogen bond with O2 (≤3Å) (Figure 1A; Table 1), and makes a close approach to O1 as well (within 3.8 Å) (Table 1). It should be noted, though, that interatomic distances vary slightly from those depicted in Table 1, depending on the individual structures under consideration. In fact, Arg-II in Cre has been suggested to interact with O1 and O2 of the scissile phosphate before strand cleavage (13). In the cleaved tyrosyl complex, the two arginines are repositioned, such that Arg-I now contacts O1 and O2, while Arg-II contacts O2 alone, in Cre as well as in Flp (Figure 1A; Table 1) (20,34). In the structure of the vanadate transition state mimic for Cre, contacts by Arg-I and Arg-II are limited to O1 and O2, respectively (15). An analogous transition state structure for Flp has not been solved.

**Table 1.**
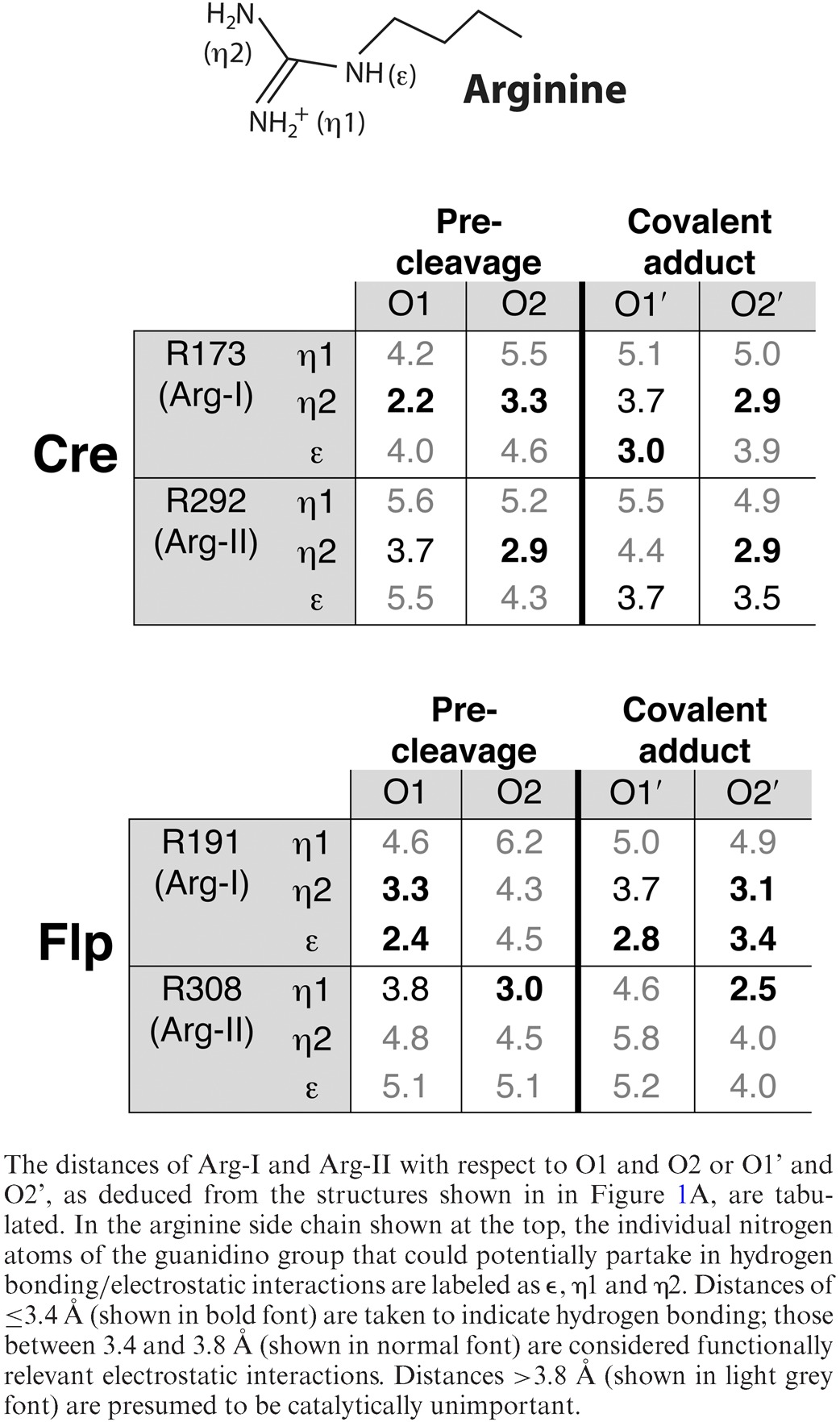
Contacts made by Arg-I and Arg-II with the scissile phosphate in the Cre and Flp active sites

From the crystallographic data, obtained using native phosphate-containing DNA, it is not possible to predict with certainty the contributions of Arg-I and Arg-II to the stereochemical course of recombination. According to the structures, depending on the stage of the reaction, Arg-I or Arg-II dispositions with respect to the non-bridging oxygen atoms are modulated. Furthermore, the static snapshots provided by the structures fail to convey the dynamic attributes of these modulations during the progression of the reaction. However, as illustrated by the experimental outcomes summarized below, these impediments can be solved (and the functional arginine-scissile phosphate interactions teased out) from the activities of recombinase variants containing only one of the arginine duo (Arg-I or Arg-II) on the scissile phosphate containing only one of the non-bridging oxygen atoms (O1, *S*_P_-MeP or O2, *R*_P_-MeP).

The focus on the interactions of Arg-I and Arg-II with the non-bridging oxygen atoms, from a stereochemical perspective, is not intended to downplay additional catalytically relevant interactions that they engage in. The transition state-mimic structures of tyrosine recombinases and type IB topoisomerases are consistent with a role for Arg-I in stabilizing the 5′-hydroxyl group, and for Arg-II in orienting the tyrosine nucleophile, during strand cleavage (15,33,36). However, as far as these latter interactions are concerned, the unfavorable catalytic effects of a missing Arg-I or Arg-II are not expected to be different for the two MeP stereoisomers.

### Cre and Flp mutants containing Arg-I alone have the same stereochemical preference as the wild type enzymes in MeP reactions

Previous analyses showed that wild type Flp has a clear preference for *S*_P_-MeP over *R*_P_ (26). Wild type Cre is only weakly active on MeP (27), making it difficult to reliably assess the extent of its stereochemical bias. Perhaps the methyl substitution causes steric clashes with one or both of the arginine side chains, thus perturbing the active site configuration. However, substitution of Arg-292 (Arg-II) by lysine is reasonably well tolerated by Cre in *in vivo* and *in vitro* recombination assays with standard DNA substrates (15). The mutant Cre is also active in MeP reactions *in vitro*. We now tested Cre(R292K), as a proxy for Cre, in *S*_P_-MeP and *R*_P_-MeP reactions. Furthermore, we tested additional mutants at the Arg-II position of Cre and Flp for their stereochemical preference. For technical reasons, the stereochemically pure MeP-half-sites employed in reactions with the Flp mutants contained a C-to-A substitution immediately 5′ to MeP (26). A similar substitution in the native Flp target site (CG to AT transversion) does not eliminate, but diminishes, its reactivity (37).

**Figure 2.**
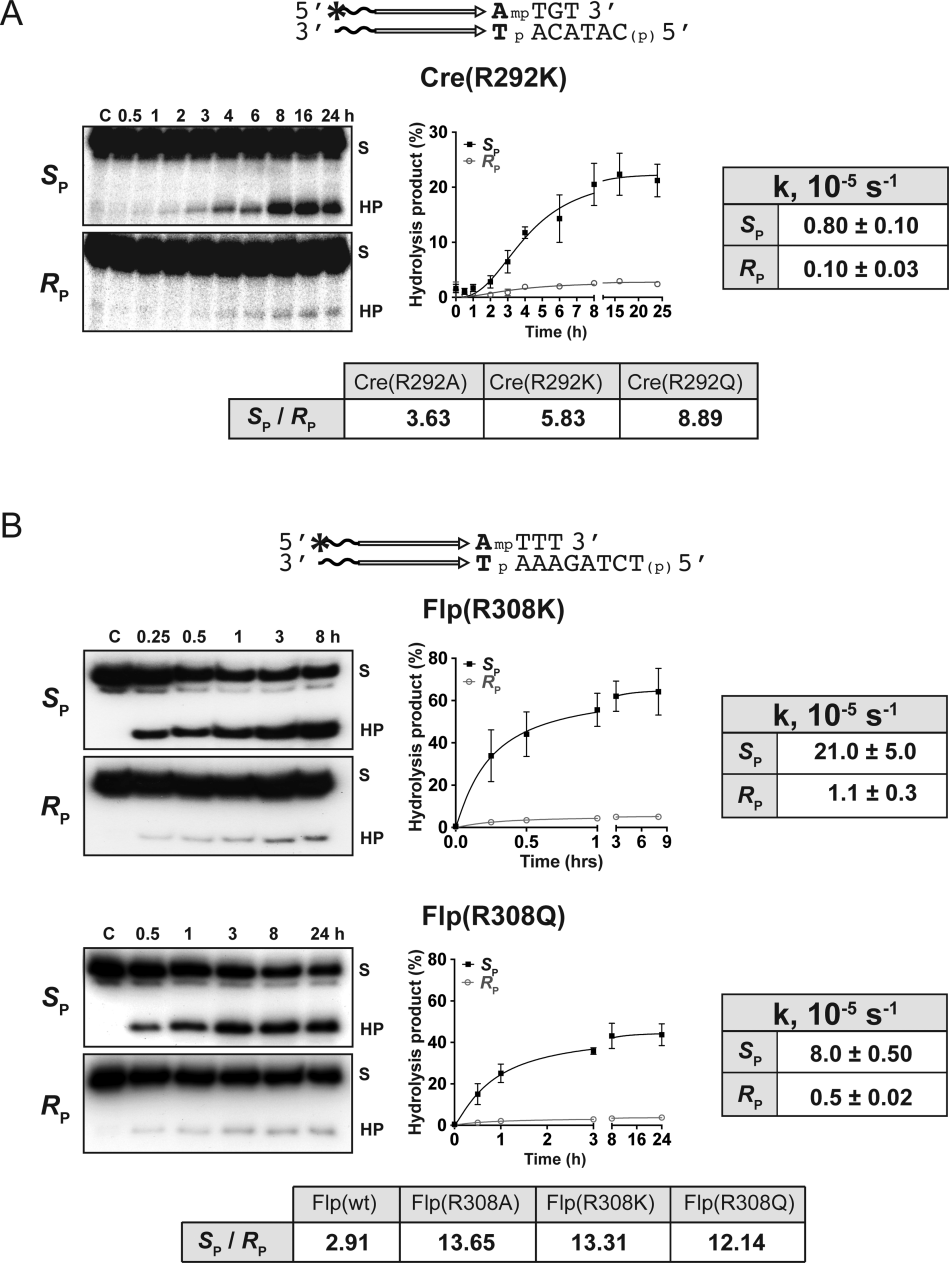
Reactions of Arg-II mutants of Cre and Flp on pure stereoisomers of MeP-substrates. (**A** and **B**) MeP-half-site substrates are schematically diagrammed above the respective reaction panels. The horizontal arrows depict the recombinase binding element, and ‘mp’ denotes the scissile MeP bond (as in Figure 1C). The wavy lines indicate extra nucleotides that are not directly relevant to the reaction. The base pair abutting the scissile MeP, highlighted in bold letters for each site, marks the terminal base pair of the Flp binding element and the immediate neighbor of the Cre binding element. The MeP-half-site for Flp contains a substitution of AT for the native CG at this position. The asterisk stands for the ^32^P-label at the 5′ end. The 5′-hydroxyl group on the bottom strand is blocked by phosphorylation. This general representation is followed in subsequent figures as well. The reactions were analyzed by phenol-chloroformn extraction, ethanol precipitation of DNA, and electrophoresis in 12% denaturing (urea) polyacrylamide gels. Any covalent recombinase–DNA intermediate would be trapped at the interphase during phenol–chloroform extraction, and would thus be excluded from the analysis. The bands labeled ‘S’ and ‘HP’ represent the substrate and the hydrolysis product, respectively. The stereochemical bias, derived as described under ‘Methods and Materials’, is listed for the indicated reactions. The value for Flp is based on published results (26) and results from similar additional reactions (data not shown). In this figure and subsequent ones, the kinetic plots represent the results, shown as the mean ± SD (standard deviation), from at least three independent assays. The pseudo-first order rate constants for the *R*_P_ and *S*_P_ reactions are listed here and in Figures 3, 4 and 6.

Arg-II mutants typified by Cre(R292K), Flp(R308K), Flp(R308Q) (Figure 2A and B) as well as Cre(R292Q) and Flp(R308A) (Supplementary Figure S2A and B) showed *S*_P_ over *R*_P_ bias, the same as wild type Flp and Cre(R292A) in previous analyses (26,27). The semi-quantitative values for the relative bias are tabulated (Figure 2A and B). Despite the weak activity of wild type Cre on MeP, its bias was also qualitatively *S*_P_-directed (data not shown).

As reported previously for Cre(R292A) (27), Cre(R292K) mediated strand cleavage using the Tyr-324 nucleophile to give the covalent intermediate, which was then slowly hydrolyzed over time (Supplementary Figure S2C). This was also the case with Cre(R292Q) (data not shown). By contrast, as noted for Flp(R308A) in earlier studies (25,26), the hydrolysis product in the Flp(R308K) and Flp(R308Q) reactions was almost all the result of direct water attack on MeP in the DNA backbone. Little or no covalent intermediate was detected in these reactions when analyzed by SDS-polyacrylamide gel electrophoresis (data not shown). The Tyr-324 dependence of the Cre mutants and the Tyr-343 independence of the Flp mutants in the MeP reaction were further verified by the loss of activity of the Cre double mutants harboring the Y324G mutation in addition to R292A, R292K or R292Q and the nearly full retention of activity by the Flp double mutants harboring the Y343G mutation in addition to R308A, R308K or R308Q (data not shown).

Thus, the native *S*_P_-MeP bias of the Cre and Flp reactions is retained, or even enhanced, when Arg-II is replaced by the positively charged lysine or by a non-charged neutral or polar residue. The stereochemistry of the strand breakage reaction is primarily determined by Arg-I through its interaction with O1. The stronger *S*_P_ bias of the Arg-II mutants of Flp than wild type Flp would be consistent with the *R*_P_ reaction being promoted by Arg-II-O2 interaction, which would be lost in the mutants. The lower bias of the corresponding Cre mutants (or comparatively higher contribution from the *R*_P_ reaction) than their Flp counterparts imply that charge stabilization at O2 relative to O1 in the absence of Arg-II is less impaired for Cre. This differential effect is consistent with the Arg-I-O2 contact seen in the Cre active site, and its absence in the Flp active site (Figure 1A; Table 1). The Cre and Flp mutants differ sharply in their utilization of the nucleophile for strand breakage, Tyr-324 by the Cre mutants and water by the Flp mutants.

### Stereochemistry of MeP reactions with Arg-I mutants of Cre and Flp: stereochemical contributions of Arg-II

Arg-I is particularly interesting in tyrosine recombination from both mechanistic and stereochemical perspectives. It is more strongly conserved than Arg-II, and conservative lysine substitutions are more or less nonfunctional in well characterized systems. Cre(R173K) does not give detectable covalent tyrosyl adduct, Holliday junction intermediate or recombinant products *in vitro* (15). Flp(R191K) retains strand cleavage potential, is strongly compromised for strand joining, and is practically inactive in recombination (38,39). As noted earlier, Arg-I in Cre and Flp is within hydrogen bonding distance of O1 of the scissile phosphate in the uncleaved DNA (Figure 1A) and in the Cre transition state mimic (15). In the cleaved intermediate formed by Cre and Flp, Arg-I retains its O1-contact, while also contacting O2 (Figure 1A). In order to probe the effects of ablating Arg-I-oxygen contacts on the stereochemistry of the reaction, we surveyed Arg-I mutants of Cre and Flp for their activities on *S*_P_-MeP and *R*_P_-MeP. The bias, or absence of bias, in these reactions can thus be attributed primarily to the relevant Arg-II-oxygen contacts.

**Figure 3.**
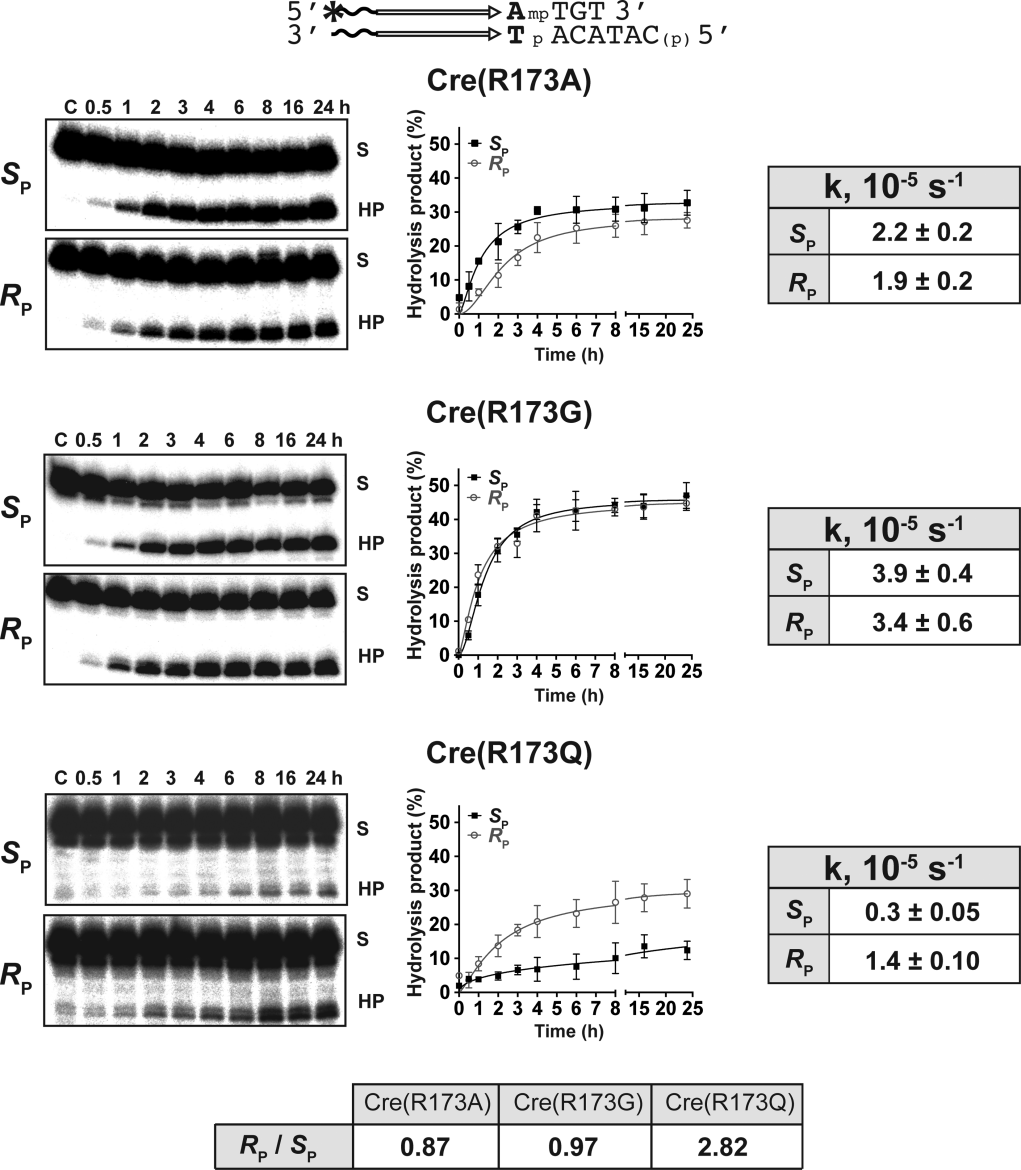
Reactions of Arg-I mutants of Cre on stereochemically pure MeP-half-sites. The analyses were performed as described under Figure 2 using the indicated MeP-half-site and the Arg-I mutants of Cre.

**Figure 4.**
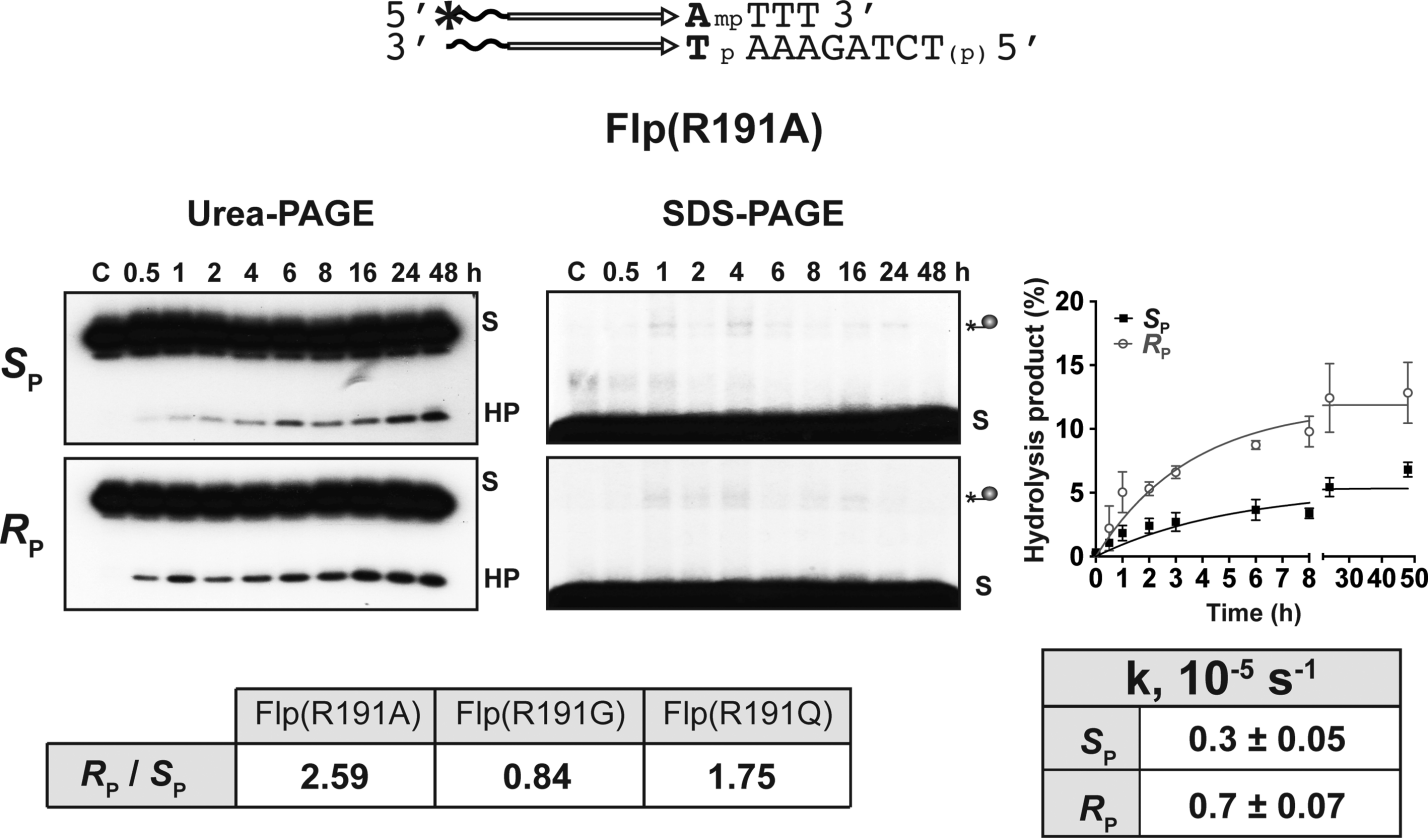
Activity of Flp(R191A) on *R*_P_ and *S*_P_ MeP-half-sites. The assays utilized pure stereoisomers of the indicated half-site. The AT base pair (shown in bold letters) adjoining the scissile MeP differs from the CG base pair present at the equivalent position in the native recombination site. The hydrolysis product was assayed by urea-polyacrylamide gel electrophoresis of DNA isolated from the terminated reactions (see legend to Figure 2). For detecting the cleaved covalent intermediate, the terminated reactions were analyzed directly by electrophoresis in 12% SDS-polyacrylamide gels.

The Arg-I mutants of Cre and Flp were distinct from the sharply *S*_P_-biased Arg-II mutants. Cre(R173A), Cre(R173G) and Flp(R191G) acted on *S*_P_ and *R*_P_ forms of MeP with almost no bias (Figure 3; data not shown). An *R*_P_ bias (2- to 3-fold) was noted for Cre(R173Q), Flp(R191A) and Flp(R191Q) (Figures 3 and 4; data not shown). As Flp(R191G) and Flp(R191Q) were weaker than Flp(R191A) in the MeP reaction (data not shown), their bias estimates were less accurate. Neither Cre(R173K) nor Flp(R191K) gave measurable activity with either MeP stereoisomer (data not shown).

It is counterintuitive that the active site accommodates alanine or glutamine at Arg-I position, but is intolerant to the conservative lysine substitution. Although lysine preserves the positive charge at this position, it could potentially promote unfavorable interactions that interfere with active site function. In *in vitro* assays on a phosphate-DNA substrate, Cre(R173H) shows limited recombination whereas no activity is detected with Cre(R173K) (15).

**Figure 5.**
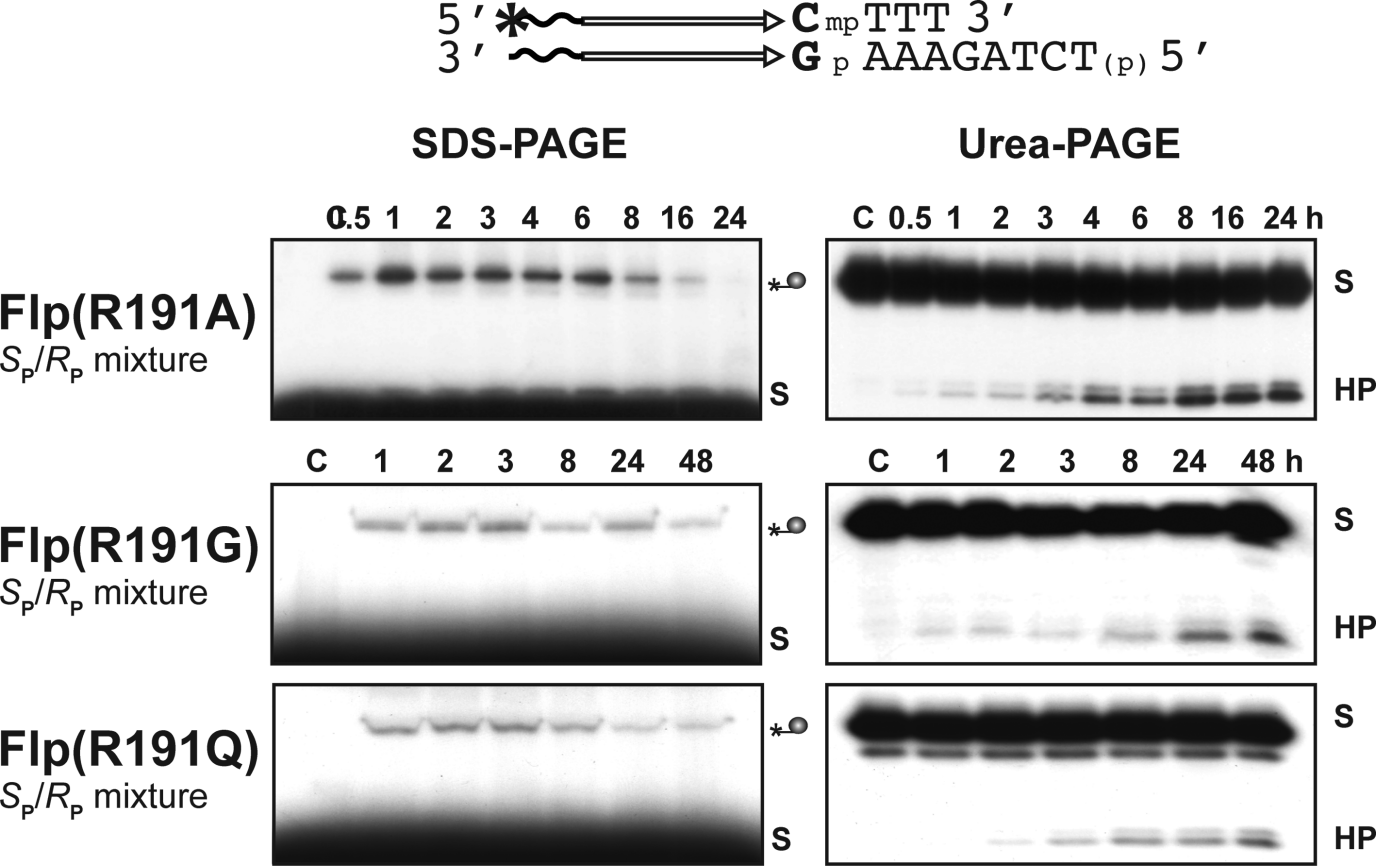
Reactions of Arg-I mutants of Flp on a racemic mixture of MeP. The assays utilized the indicated MeP-half-site as a racemic mixture. Note that this half-site harbors a CG base pair next to the scissile MeP. By contrast, the corresponding base pair is AT in the *R*_P_ and *S*_P_ MeP-half-sites used for the reactions shown in Figure 4. The covalent DNA–protein adduct was probed by 12% SDS-polyacrylamide gel electrophoresis (as described under Figure 4).

In the Cre Arg-I mutant reactions with individual MeP stereoisomers or their racemic mixture, the cleaved covalent intermediate appeared at early time points, and its amounts decreased as the hydrolysis product built up (Supplementary Figure S3; data not shown). This pattern is consistent with their precursor–product relationship. However, no covalent intermediate was detected with Flp(R191A), Flp(R191G) or Flp(R191Q) in the *S*_P_-MeP or *R*_P_-MeP reactions (Figure 4; data not shown), suggesting that the reaction was comprised almost wholly of direct hydrolysis. This result would seem to contradict the previous observation that Flp(R191A) reaction with the MeP racemic mixture yields the hydrolysis product mainly via the covalent intermediate (25). When tested on the same racemic mixture, Flp(R191G) and Flp(R191Q) gave the tyrosyl intermediate early in the reaction, followed subsequently by the hydrolysis product (Figure 5). Note that the racemic mixture of MeP differs from the stereochemically pure forms in the base flanking MeP at its 5′-side, the native C in the former replaced by A in the latter. The mechanistic difference in the reactions of the Arg-I mutants of Flp, depending on the substrate context, suggests that the nature of the 5′-base adjoining the scissile phosphate can sway the reaction towards tyrosine-mediated strand cleavage or towards direct hydrolysis. This aspect is more carefully examined in experiments described below (see Figure 7 and Supplementary Figure S5).

**Figure 6.**
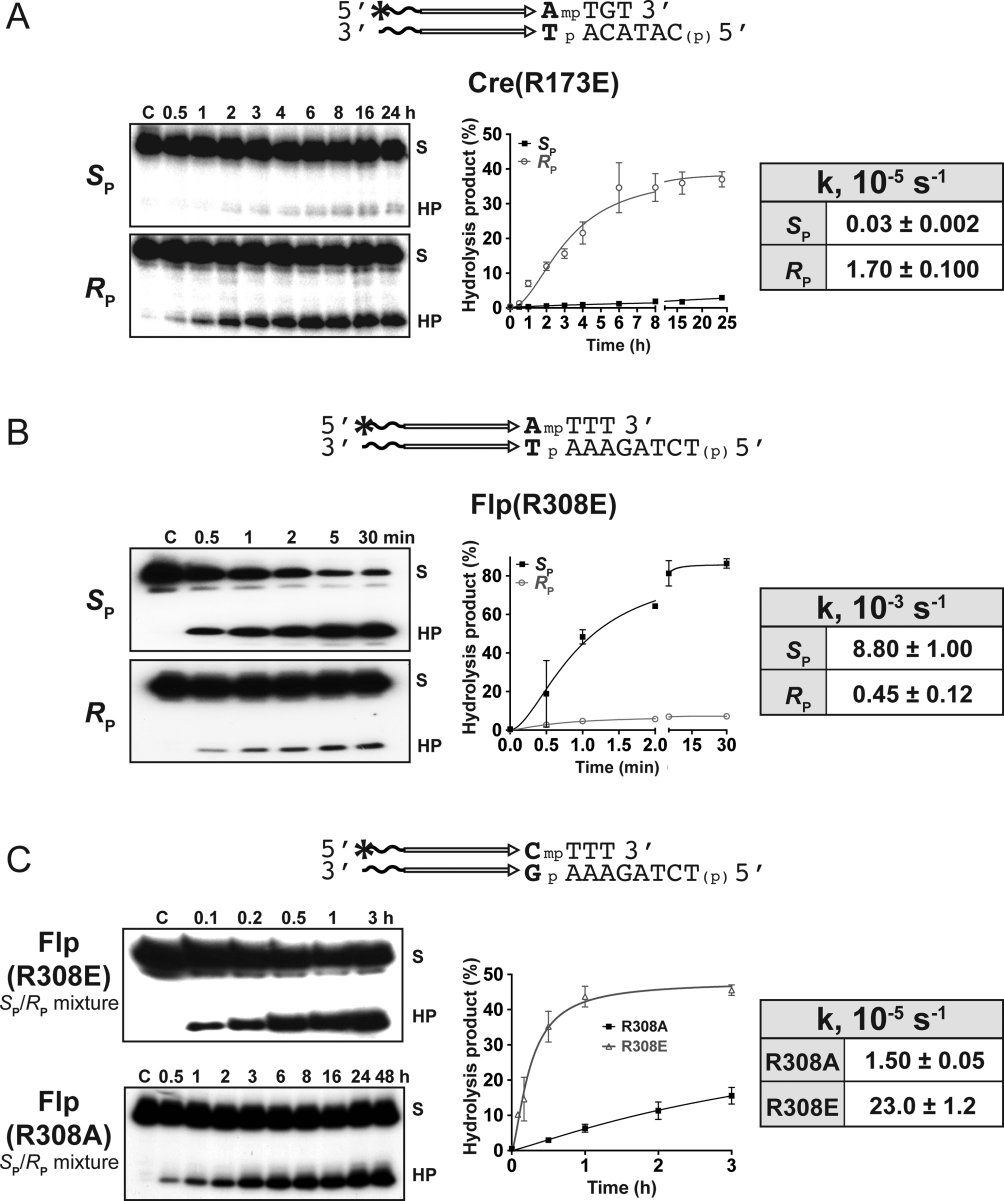
Activities and stereochemical preferences of Cre(R173E) and Flp(R308E) in MeP-half-site reactions. The reactions were performed on stereochemically pure forms of MeP (**A** and **B**) or a racemic mixture of MeP (**C**). The half-site in the stereospecific reaction with Flp(R308E) contained the base A as the 5′-neighbor of MeP in place of the native base C. The results for Flp(R308A) (**C**) are taken from published work (26), and are shown here to highlight the enhanced activity of Flp(R308E).

**Figure 7.**
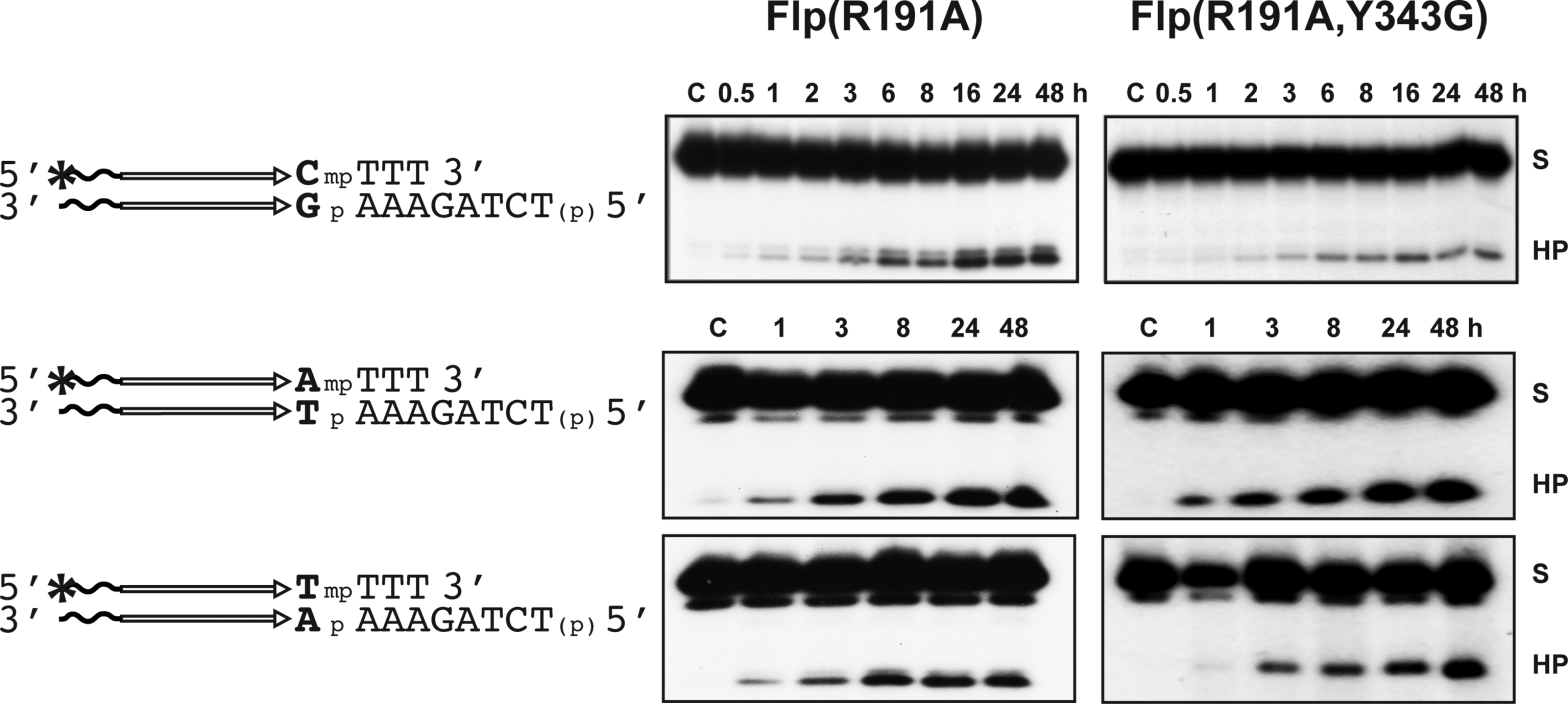
The effect of the base immediately 5′-adjacent to MeP on strand breakage by the tyrosine nucleophile versus water. The reactions utilized racemic mixtures of the indicated MeP-half-sites. Flp(R191A) and Flp(R191A, Y343G) were tested against each of the three half-sites under the same reaction conditions and at constant protein-to-DNA molar ratios.

The near absence of stereochemical bias (or *R*_P_ bias) displayed by the Arg-I mutants is consistent with the O2 contact made by Arg-II, and its proximity to O1 as well, in the uncleaved DNA in Cre and Flp structures (Figure 1A; Table 1). In addition, as suggested by the Cre structure of the transition state mimic, the conserved active site histidine (His-289 in Cre; His-305 in Flp) may assist the *S*_P_-MeP reaction by hydrogen bonding to O1 (15).

### Charge reversal at Arg-I or Arg-II has reverse effects on Cre and Flp in MeP reactions

Attack of the backbone MeP by water, with almost complete suppression of tyrosine-mediated strand cleavage, was first noticed for Flp(R308A) and Flp(R308A, Y343F) (26). Direct MeP hydrolysis constitutes only a minor reaction in the case of Flp(R191A) (25). In contrast to Flp, lack of Arg-173 or Arg-292 does not elicit direct MeP hydrolysis by Cre (27,28). It was suggested that the Arg-308 side chain in Flp electrostatically misdirects the abundant water nucleophile, thus preventing an aberrant reaction that aborts recombination and induces DNA damage. In an analogous fashion, the phosphotyrosyl intermediate formed by vaccinia topoisomerase appears to be protected against hydrolysis by the negative charge on the scissile phosphate (40,41). If the protective role of Arg-II is dependent on its positive electrostatics, a negatively charged side chain at this position might be as ineffective as the uncharged alanine side chain, might aggravate the direct hydrolysis reaction, or even destroy active site function entirely. We therefore compared the effects of glutamic acid substitution at Arg-I or Arg-II in Cre and Flp on the MeP reaction.

Cre(R173E) was active, formed the tyrosyl intermediate before promoting its hydrolysis, and was highly biased towards *R*_P_ (Figure 6A; Supplementary Figure S4A). Cre(R292E) was nearly inactive (Supplementary Figure S4A). The situation was almost reversed with Flp. Flp(R308E) showed potent hydrolytic activity with a strong *S*_P_ bias (Figure 6B). The action of Flp(R191E) on an MeP-half-site racemic mixture (with C as the 5′ neighbor of MeP) revealed faint bands of the cleaved tyrosyl intermediate, whose further hydrolysis was undetectable (Supplementary Figure S4B). Flp(R308E) was at least an order of magnitude faster than Flp(R308A) in the kinetics of hydrolysis of a racemic mixture of the MeP half-sites (Figure 6C). The pronounced kinetic difference between these two mutants was even more accentuated in their reactions with the stereochemically pure MeP substrates (Figure 6B; Supplementary Figure S2B). Approximately half the racemic MeP mixture was converted to product by Flp(R308E) in ∼30 min, while the conversion of the *S*_P_ form MeP was ∼90% in the same duration (Figure 6B, C). These yields are as expected for the marked difference in the reactivities of the *R*_P_ and *S*_P_ forms in the racemic mixture. No covalent intermediate was detected with Flp(R308E) in the reactions with pure MeP stereoisomers or with a racemic mixture (data not shown).

Introduction of negative charge at the Arg-I position in Cre does not appear to impede Arg-II contact with O2, as evinced by the *R*_P_-biased activity of Cre(R173E). Conversely, a similar charge replacement at the Arg-II position in Flp seems not to disrupt the Arg-I-O1 contact, thus accounting for the *S*_P_-biased strong hydrolytic activity of Flp(R308E). However, negative charge at the Arg-II position in Cre and at Arg-I position in Flp likely obstructs Arg-I-O1 and Arg-II-O2 contacts, respectively, rendering Cre(R292E) and Flp(R191E) inactive on MeP-substrates.

As noted earlier in the comparison between Cre(R173K) and Cre(R173A) or Cre(R173Q), the ability of Cre(R173E) to perform *R*_P_-biased strand cleavage in the MeP substrate, contrasted by the inactivity of Cre(R173K), could not have been anticipated. The inadmissibility of lysine in place of arginine at position 173 is thus most simply attributed to the induction of non-conducive interactions and/or the disruption of pertinent ones by lysine.

Glutamic acid is only one of an array of amino acids tolerated at position 308 of Flp, all of which, however, alter the chemistry of MeP cleavage by switching from tyrosine to water as the cleavage nucleophile. Conceivably, the choice of Arg-II in the Flp active site must have been dictated not only by the need to stabilize the negative charge at O2 but also by the constraint of utilizing the Tyr-343 nucleophile, to the exclusion of other potential nucleophiles, during strand cleavage.

### Channeling of Flp–MeP reaction into the tyrosyl intermediate or directly into the hydrolytic product is determined by the base neighboring the scissile MeP

In the native Flp target site, the scissile phosphate at either end of the strand exchange region is flanked by the terminal CG base pair of the Flp binding element. This position accepts TA with little or no loss of recombination activity, and AT with a roughly 5-fold reduction in activity (37). However, GC is unacceptable, reducing recombination by >100-fold. The CG to GC transversion is highly detrimental to both the Flp binding and catalytic steps (42).

In the MeP-half-site containing CG as the 5′-neighbor of MeP, reaction by wild type Flp or Flp(R191A) proceeds mainly via the tyrosyl intermediate (25,26). Direct hydrolysis accounts for only ∼25% of the product yield (see also Figure 7 and Supplementary Figure S5). However, in Flp and Flp(R191A) reactions with AT as the 5′-flanking neighbor of MeP, the tyrosyl intermediate is greatly reduced or is barely detectable (26) (Figure 4). The non-native AT base pair next to the scissile MeP appears to modulate the reaction mechanism, favoring direct hydrolysis at the expense of tyrosine-mediated cleavage. In order to more critically examine this aspect, we tested Flp, Flp(R191A) and the corresponding single and double mutants harboring the Y343G mutation against MeP-half-sites containing each of the four base pairs adjoining MeP. The wild type Flp and Flp(R191A) reactions report on the combined yields from tyrosine-mediated cleavage and direct hydrolysis, while the Flp(Y343G) and Flp(R191A, Y343G) reactions convey the extent of direct hydrolysis alone.

The reactions depicted in Figure 7 and Supplementary Figure S5 were performed with racemic mixtures of the indicated MeP-half-sites. In contrast to the (CG)-MeP-substrate, the (AT)- and (TA)-substrates gave nearly 1:1 amounts of the hydrolysis product with Flp(191A) and Flp(R191A, Y343G) (Figure 7). The equivalence of the reaction in the presence or absence of Tyr-343 indicates that the tyrosyl intermediate was bypassed in the formation of the hydrolysis product from the (AT)- and (TA)-substrates. The product ratio with the (CG)-substrate was roughly 3:1 in favor of Flp (R191A), suggesting that, in this reaction, tyrosine-mediated cleavage distinctly outperformed direct hydrolysis. Concordant results were obtained by performing (AT)-, (TA)- and (CG)-MeP reactions in the presence of wild type Flp or Flp(Y343G) (Supplementary Figure S5). Consistent with the earlier mutational analysis of the native recombination site (37,42), the (GC)-MeP-substrate gave no detectable product with Flp or with any of the single or double mutants (data not shown).

Thus, the presence of a non-native base pair as the 5′-neighbor of MeP blocks the reaction or diverts it from productive recombination towards futile hydrolysis. It is not clear to us how the cleavage nucleophile can be altered depending on the chemical nature of a base (or a base pair). However, the Flp active site is adept at activating a variety of nucleophiles for phosphoryl transfer under distinct reaction contexts. These nucleophiles include, in addition to tyrosine mimics, chemically unrelated agents: hydrogen peroxide, glycerol and vicinal 2′-hydroxyl groups from ribonucleotides placed at specific positions in DNA substrates (43–47). It has been suggested that the native CG neighboring the scissile phosphate in the Flp target site may assist binding/catalysis via the hydrogen bonding interaction between N-7 of guanine and Lys-82 of Flp (42). An equivalent hydrogen bond is feasible in the CG to TA transition but not in the CG to GC or AT transversions. The loss of specific DNA–protein interactions or the spawning of altered interactions may awaken aberrant catalytic tendencies within the Flp active site.

## DISCUSSION

**Figure 8.**
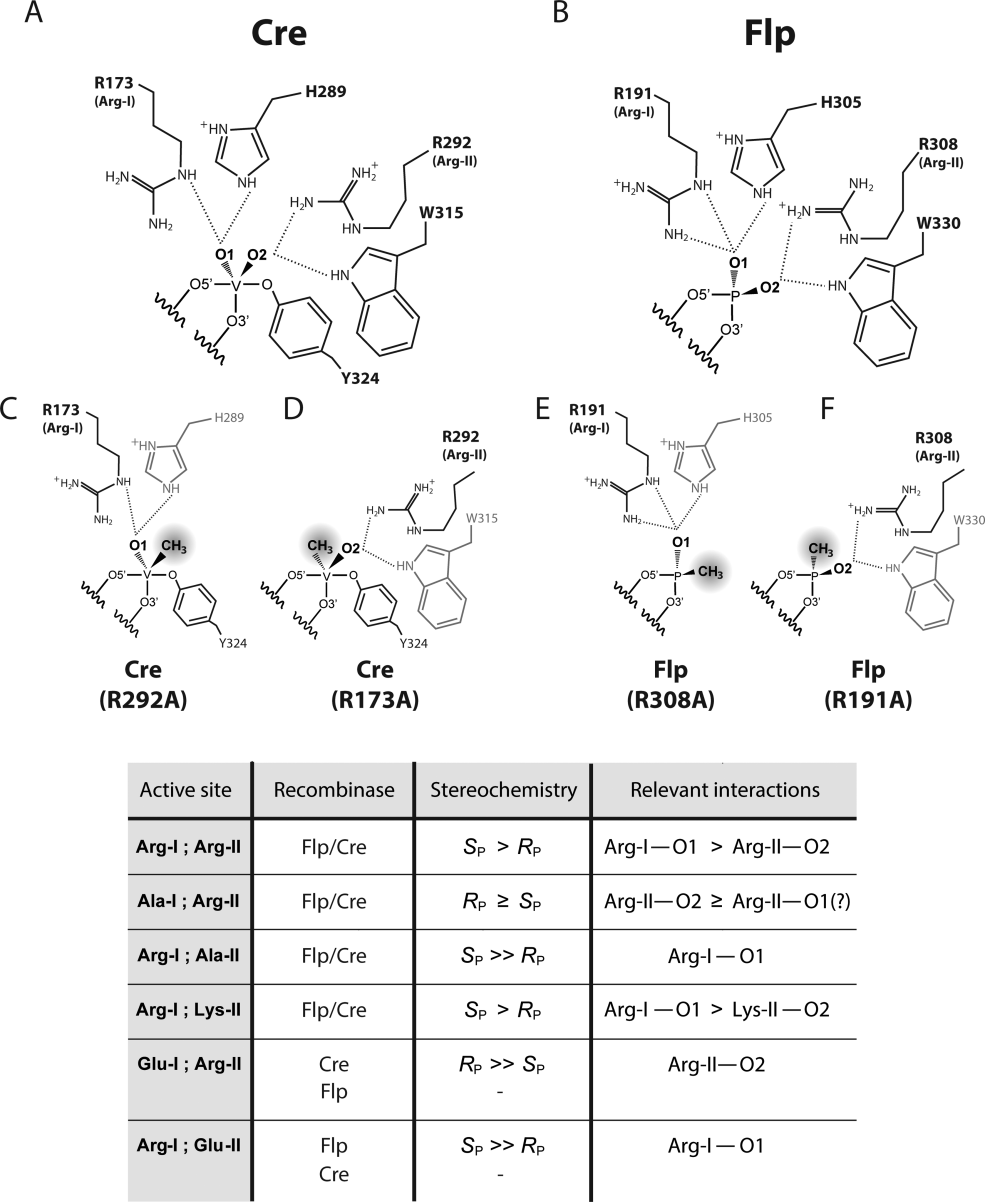
Stereochemistry of the Cre and Flp active sites revealed by *S*_P_- and *R*_P_-MeP reactions. The contacts made by the conserved arginines (Arg-173 and Arg-292), histidine (His-289) and tryptophan (Trp-315) of Cre with the scissile phosphate in the transition state mimic (15) are displayed at the top left (**A**). The corresponding contacts made by Arg-191, Arg-308, His-305 and Trp-330 of Flp with the scissile phosphate in the uncleaved DNA (34) are depicted at the top right (**B**). Shown below are the contacts presumed to be retained by the Arg-I and Arg-II point mutants of Cre in the MeP transition state (**C**, *S*_P_-MeP; **D**, *R*_P_-MeP) and by the corresponding mutants of Flp with the scissile MeP before cleavage (**E**, *S*_P_-MeP; **F**, *R*_P_-MeP). The results from a selected relevant set of MeP reactions are tabulated. The inferred Arg-I and Arg-II interactions with the non-bridging oxygen atom(s) consistent with the observed stereochemistry of each reaction are summarized. The potential stereo-specific contributions by the conserved histidine and tryptophan (His-289 and Trp-315 in Cre; His-305 and Trp-330 in Flp) have not been tested in this study by mutational analysis. Some of the Arg-I mutants of Cre and Flp exhibit, in addition to their activity on *R*_P_-MeP, considerable activity on *S*_P_-MeP as well, suggesting that the electrostatic effect of Arg-II may spread to O1. Potential interaction of the conserved histidine with O1 may also assist the *S*_P_ reaction. All of the Cre mutants active on MeP give the hydrolysis product via the tyrosyl intermediate; none mediate direct hydrolysis. Flp and Arg-I mutants of Flp active on MeP promote formation of the tyrosyl intermediate in a half-site containing C as the immediate 5′-neighbor of MeP. They mediate direct hydrolysis much less efficiently. Replacing this C by A or T steers the reaction sharply towards direct hydrolysis. Arg-II mutants of Flp yield the hydrolysis product with little or no tyrosyl adduct formed as an intermediate. No activity or extremely weak activity is denoted by the ‘—’ symbol.

The present analyses of Cre and Flp recombinases provide a stereochemical view of the tyrosine recombinase active site in action. They also shed new light on the mechanistic contributions of its highly conserved Arg-I and Arg-II residues. These insights were garnered from reactions promoted by active sites lacking either Arg-I or Arg-II on stereochemically pure MeP-substrates harboring only O1 or O2. Our findings set the stage for examining the potential roles of the conserved histidine and tryptophan of Cre and Flp active sites in fine-tuning the stereochemical effects of Arg-I and Arg-II. In the Cre transition state mimic, His-289 and Trp-315 contact O1 and O2, respectively (13,15). The principal findings from this study and their implications are summarized in Figure 8, and are discussed below.

Since the structures of MeP-DNA complexes formed by Cre or Flp have not been solved, we have interpreted the biochemical results from MeP reactions within the general framework of Cre and Flp structures in association with phosphate-containing DNA. Furthermore, we assume that the potential perturbations of the active site caused by the MeP modification are rather small, and also similar between Cre and Flp. These assumptions are reasonable, given the nearly identical organization of their active site residues around the scissile phosphate as well as the relatively small increase of ∼30% in the Van der Waals radius of the methyl group (∼200 pm) compared to the oxygen atom (∼150 pm) (48,49). For comparison, the methyl group is only slightly larger than the sulfur atom (Van der Waals radius of ∼185 pm) replacing one of the non-bridging oxygen atoms in phosphorothioate, a widely used phosphate analog. The P-S bond length in phosphorothioate (1.934 Å) (50,51) is greater than the P–C bond length in MeP (1.80 Å) (52). The P–O (non-bridging) distances in phosphorothioate (1.501 Å) (50,51) and in unmodified phosphodiesters (1.483 Å) (50) are similar to the P=O (non-bridging) distance in MeP (1.49 Å) (52).

MeP modification in DNA has so far been employed profitably, though it is still underutilized, in the analyses of topoisomerase and recombinase reactions (25–28,40,41). By introducing MeP at several individual positions in the scissile and non-scissile strands of the vaccinia topoisomerase target site, Shuman *et al*. were able to separate the contributions towards strand cleavage by simple electrostatic interactions from those by stereospecific polar interactions with the non-bridging oxygen atoms (53). Tapping the full potential of MeP substitution in exploring the variety of biologically important nucleotidyl transfer mechanisms, DNA transposition and intron mobility (2,54), for example, is an exciting future prospect.

### Stereochemical features of Cre and Flp active sites

Arg-I-O1 contact is the principal determinant of the *S*_P_-MeP preference of wild type Cre and Flp. Their structures containing uncleaved DNA reveal this contact, which is also present in the vanadate transition state-mimic formed by Cre (15,34,35). A similar *S*_P_ preference has been demonstrated for the vaccinia topoisomerase, and is independent of the arginine corresponding to Arg-II (41). The active sites of tyrosine recombinases and type IB topoisomerases in general are likely to conform to these stereochemical features.

The lack of bias or the *R*_P_ bias of the Arg-II assisted MeP reactions is consistent with the O2-contact made by Arg-II as well as its relative proximity to O1, enabling it to extend its electrostatic reach to this non-bridging atom. Furthermore, the conserved histidine may also play a role in charge stabilization at O1.

### MeP reaction is sustained by Arg-II (but not Arg-I) in Cre and by Arg-I (but not Arg-II) in Flp when the partner arginine is replaced by glutamic acid

The relatively strong (and *R*_P_-biased) activity of Cre(R173E) on MeP is contrasted by the near inactivity of Flp(R191E). In a reversal of this situation, Flp(R308E) is the most potent of all the Flp mutants tested (with *S*_P_ bias), while Cre(R292E) is among the least active of the Cre mutants. Despite their apparent strong positional conservation, the stereochemical roles of Arg-I and Arg-II are not correspondingly conserved in Cre and Flp. The influx of negative charge from glutamic acid substitution of Arg-I disrupts charge stabilization at O1 in Cre (R173E) (effected by Arg-I perhaps with collaboration from His-289), but not at O2 (promoted by Arg-II). However, both O1 and O2 are affected in Flp(R191E). The analogous build-up of negative charge from placing glutamic acid at the Arg-II position leaves Arg-I-O1 contact and the *S*_P_ reaction unaffected in Flp (R308E). In Cre(R292E), the altered electrostatic effect encompasses O1 and O2, rendering Cre(R292E) inactive on *S*_P_-MeP and *R*_P_-MeP. In the phosphate transition states of these recombinases, the combined electrostatic effects of Arg-I and Arg-II are designed to serve the same catalytic end. Yet, as revealed by their MeP reactions, the contributions of the individual arginines to the electrostatic ambiance at O1 and O2 are distinct between Cre and Flp.

### The MeP-hydrolysis activity of Arg-I and Arg-II mutants of Flp: the effect of the 5′-neighboring base

Mutations at the Arg-II position induce a strong MeP-hydrolytic activity in Flp, which almost completely outcompetes strand cleavage by the Tyr-343 nucleophile (26) (this study). MeP-hydrolysis is only a minor side reaction with wild type Flp or Arg-I mutants of Flp, as long as the 5′-neighbor of MeP is C (as is the case in the native recombination target site) (25) (this study). These findings are consistent with Arg-II of Flp electrostatically protecting MeP from attack by water. In the vanadate transition state mimics of Leishmania and poxvirus topoisomerases and Cre, Arg-II forms a hydrogen bonding interaction with the phenolic oxygen of the tyrosine nucleophile (15,33,36). Assuming the same interaction obtains in Flp, the loss of Arg-II may misposition the tyrosine, exposing MeP to attack by water. However, this possibility is unlikely. The direct hydrolytic activities of Flp(Y343G) and Flp(R191A, Y343G) (altogether lacking the tyrosine side chain) are more or less similar, but much lower than that of Flp mutants lacking Arg-308, Flp(R308A) or Flp(R308E) (25,26) (this study). Furthermore, Arg-II mutants of Cre do not mediate direct MeP hydrolysis.

The surprisingly strong enhancement of MeP hydrolysis by the R308E mutation would be consistent with Glu-308 directly participating in catalysis. It may act as a general acid to potentiate MeP-attack by water or by the hydroxide anion. The substitution of the active site tyrosine by glutamic acid induces an endonuclease activity in vaccinia topoisomerase against a standard phosphate-containing DNA substrate (55). It is not clear whether this reaction is the result of direct water attack, or proceeds via a covalent acyl intermediate. Although the endonuclease activities of Flp(R308E) and Topo(Y274E) may not be directly comparable, they have general implications in the evolution of bio-catalysis. It is almost axiomatic that, once an active site has been established for a certain biological reaction, it may be refashioned to perform chemically related, but mechanically distinct, reactions. The modifications required to elicit some of these functional transitions may be relatively simple. The ability of Flp to employ water or the 2′-hydroxyl group as nucleophiles for strand scission (26,47) (this study) may speak to the potential evolution of the recombinase active site from an elementary nuclease active site, perhaps via a topoisomerase active site.

The shift from tyrosine-mediated cleavage to almost exclusively direct hydrolysis by Flp or Flp(R191A) can also be elicited by switching the 5′-base neighboring the scissile position from the native C to A or T in the MeP substrate. Viewed in the context of the similar effect of the Arg-308 mutations, with any of the three bases (C, A or T) as the 5′-neighbor of MeP, these findings suggest a potential guiding principle in the catalytic design of the recombinase active site. Not only is it important to optimize substrate interactions that foster the desired chemical steps, but it is also equally important to exclude those interactions that promote wasteful side reactions resulting in DNA damage.

### The self-contained active site of Cre and the shared active site of Flp

Both Cre and Flp are monomers in solution and bind to DNA as monomers. Occupancy of a native target site by two recombinase monomers triggers their dimerization, and association of two target-bound dimers yields the recombination synapse. At least some of the differences between Cre and Flp in the half-site reactions may result from a Cre monomer being self-sufficient, and a Flp monomer being dependent on a neighboring monomer, for active site assembly (20–22,56). However, allosteric stimulation of the Cre active site by the interaction between two adjacent Cre monomers is possible.

The half-site substrates employed in our assays can be bound only by a single Cre or Flp monomer. The assembly of the shared Flp active site, required for tyrosine-mediated strand cleavage in a half-site, is still possible, as the Flp-bound half-sites can associate to form dimers, trimers and tetramers (31). Analogous higher-order complexes are presumably formed by the Cre-bound half-sites as well. Direct MeP hydrolysis exhibited by the Arg-II mutants of Flp may result from the opportunity for water to access the activated MeP in a half-site bound by a mutant monomer before it is engaged by the tyrosine nucleophile from a second monomer (26). Such a predicament is avoided during Cre reactions as the binding of a Cre monomer to a half-site and the engagement of the scissile MeP by the tyrosine nucleophile would be more or less concomitant events.

## SUMMARY AND CONCLUSIONS

The outcomes of the MeP reactions analyzed in the present study are consistent with the stereochemical course of Cre and Flp recombination being primarily determined by the conserved active site residues Arg-I and Arg-II. The *S*_P_ over *R*_P_ preference of the wild type recombinases suggest that Arg-I–O1 interaction is dominant over Arg-II–O2 interaction in specifying the stereochemistry of the native reaction. Consistent with this inference, the MeP reactions assisted by Arg-I alone is strongly *S*_P_-biased, while those supported by Arg-II alone can be nearly bias-free or *R*_P_ biased. The stereochemical insights gained from the MeP reactions can be rationalized in terms of the active site-scissile phosphate interactions seen in structures containing the uncleaved DNA or the vanadate transition state mimic. The potential roles of the conserved histidine (His-289 in Cre; His-305 in Flp) and tryptophan (Trp-315 in Cre; Trp-330 in Flp) in fine-tuning the Arg-I and Arg-II stereochemical effects through general electrostatic or specific polar interactions are yet to be investigated.

Against this overall stereochemical conformity between Cre and Flp, the MeP reactions also reveal clear distinctions between the two. Glutamic acid at the Arg-I position is permissive for MeP bond breakage by Cre, but not by Flp, while glutamic acid at the Arg-II position has the opposite effect. The Arg-I as well as Arg-II mutants of Cre utilize the active site tyrosine nucleophile to bring about MeP cleavage. The Arg-I mutants of Flp utilize the tyrosine nucleophile predominantly, while the Arg-II mutants of Flp utilize water nucleophile almost exclusively. Wild type Flp or the Arg-I mutant Flp(R191A) switches to water nucleophile when the terminal base pair of the Flp binding element abutting the scissile MeP is changed from the native CG to AT or TA. Collectively, these findings bring to light two attributes of the tyrosine recombinase active site. Although the combined catalytic contribution by Arg-I and Arg-II is well conserved between Cre and Flp, the individual contributions of Arg-I and Arg-II are not equivalent in the two recombinases. The inherent capacity of the Flp active site to utilize errant nucleophiles antithetical to recombination is curtailed by appropriate chemical choices within the active site and in the DNA substrate.

## SUPPLEMENTARY DATA

Supplementary Data are available at NAR Online.

SUPPLEMENTARY DATA
