# Supplementary material for: Stereospecific suppression of active site mutants by methylphosphonate substituted substrates reveals the stereochemical course of site-specific DNA recombination
Source: Nucleic Acids Res. 2015 May 20;43(12):6023–37. doi: 10.1093/nar/gkv513 (PMC4499138; doi:10.1093/nar/gkv513)
Supplement: SUPPLEMENTARY DATA [file supp_43_12_6023__index.html]

Stereospecific suppression of active site mutants by methylphosphonate substituted substrates reveals the stereochemical course of site-specific DNA recombination — Stereospecific suppression of active site mutants by methylphosphonate substituted substrates reveals the stereochemical course of site-specific DNA recombination — SUPPLEMENTARY DATA 

# Stereospecific suppression of active site mutants by methylphosphonate substituted substrates reveals the stereochemical course of site-specific DNA recombination

## SUPPLEMENTARY DATA

- SUPPLEMENTARY DATA
