# Supplementary material for: Stereospecific suppression of active site mutants by methylphosphonate substituted substrates reveals the stereochemical course of site-specific DNA recombination
Source: Nucleic Acids Res. 2015 May 20;43(12):6023–37. doi: 10.1093/nar/gkv513 (PMC4499138; doi:10.1093/nar/gkv513)
Supplement: SUPPLEMENTARY DATA [file supp_gkv513_nar-00566-h-2015-File011.pdf]

SUPPLEMENTARY DATA & FILES FOR:

Stereospecific suppression of active site mutants by methylphosphonate substituted substrates reveals the stereochemical course of site-specific DNA recombination

*Paul A Rowley[1], *Aashiq H Kachroo[1], Chien-Hui Ma[1], Anna D Maciaszek[2], Piotr Guga[2] and Makkuni Jayaram[1]

* contributed equally

[1]Department of Molecular Biosciences,

University of Texas at Austin,

Austin, TX 78712

and

[2]Centre of Molecular and Macromolecular Studies,

Polish Academy of Sciences,

Department of Bioorganic Chemistry,

Sienkiewicza 112, 90-363 Lodz, Poland

Corresponding author: Makkuni Jayaram,

Tel: 512-471-0966

E. mail: jayaram@austin.utexas.edu

Table of contents:

Figures & Figure legends S1 to S5

Table S1.

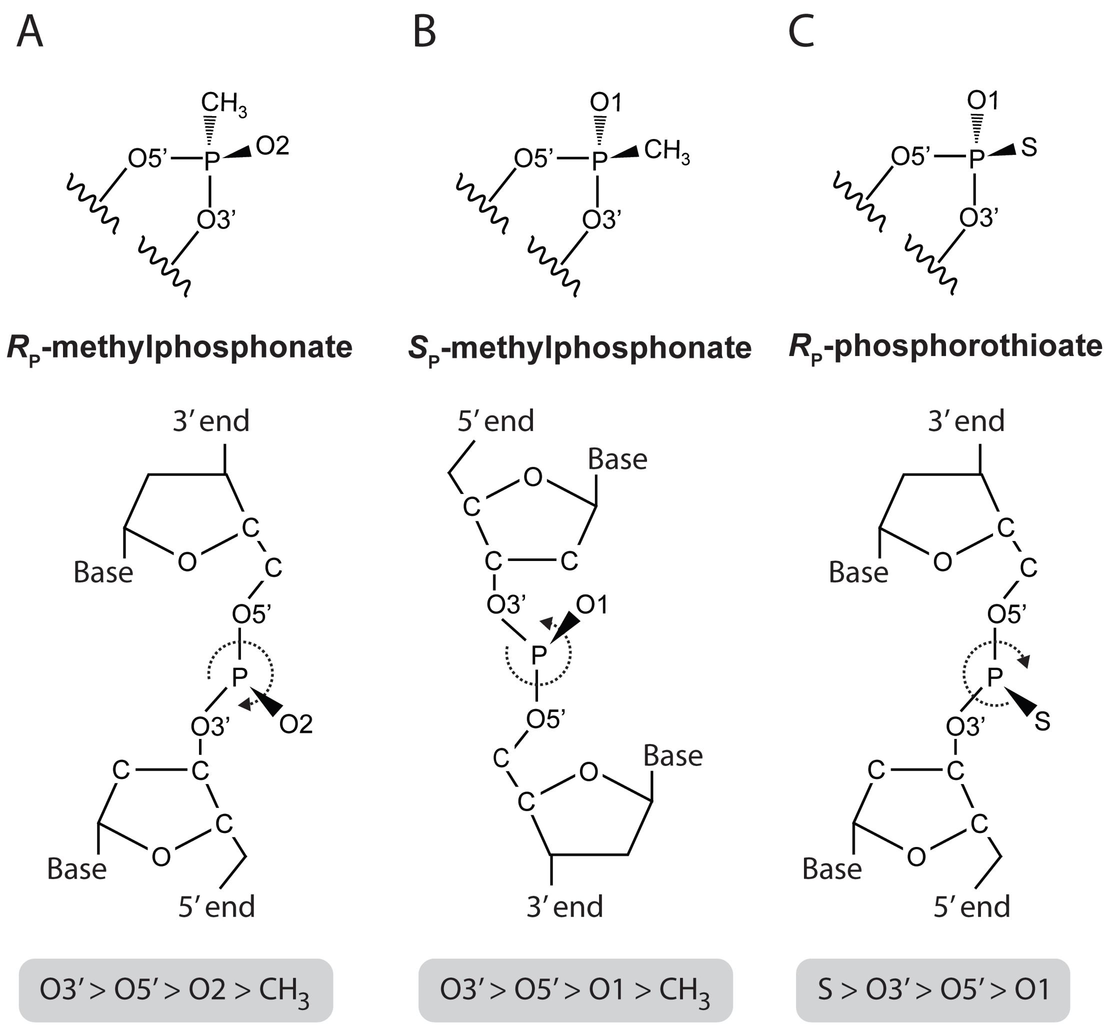

Figure S1

Figure S1. Phosphodiester stereochemistry: chirality resulting from the replacement of a non-bridging oxygen atom by the methyl group or by sulfur. The structures of the R_P and S_P stereoisomers of methylphosphonate (MeP; see also Figure 1B) and of R_P-phosphorothioate are shown in the top panels (**A-C**). The assignment of chirality by the Cahn-Ingold-Prelog (CIP) rules is depicted below each structure. In **A** and **B**, the lowest priority methyl group is placed behind the plane of the paper. The other three groups are arranged, according to their priorities (O3' > O5' > O2), in a clockwise fashion in the R_P form of MeP (**A**). The arrangement (O3' > O5' > O1) is

counterclockwise in the S_P form of MeP (**B**). The bridging and non-bridging oxygen atoms take precedence over the carbon atom of the methyl group by their higher atomic number. Between the two bridging oxygen atoms, the 3'-oxygen has higher priority than the 5'-oxygen. Note that the 3'-oxygen is bonded to a carbon that, in turn, is bonded to two other carbon atoms. The carbon atom linked to the 5'-oxygen is bonded to only one carbon atom. The non-bridging oxygen atom has lower priority than the bridging oxygen atoms even though it is double bonded. According to the stereochemical convention, when a tetrahedral atom at the stereocenter is linked to four ligands, each of the four bonds is treated as a single bond. In the phosphorothioate (**C**), the sulfur atom supersedes the other three groups (S > O3' > O5' > O1). The clockwise arrangement of S-O3'-O5' assigns this stereoisomer as R_P. The opposite assignments for O2 substitution in MeP (S_P) (**B**) and phosphorothioate (R_P) (**C**) is the consequence of the opposite priorities of the methyl group and sulfur with respect to the replaced oxygen atom.

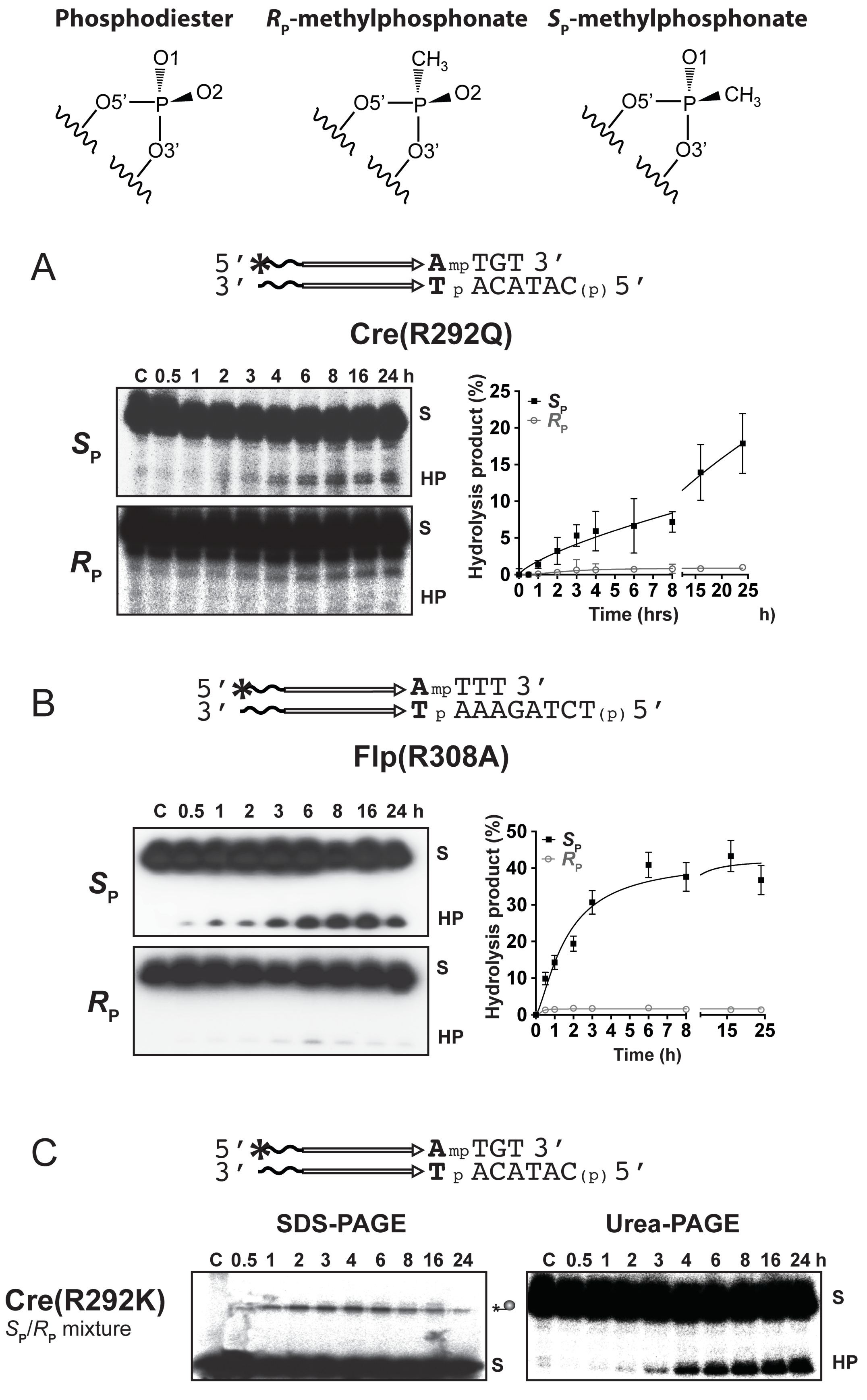

Phosphodiester
RP-methylphosphonate
SP-methylphosphonate
O1
O2
O5'
P
O3'
CH3
A
5' AmpTGT 3'
3' Tp ACATAC(p) 5'
Cre(R292Q)
C 0.5 1 2 3 4 6 8 16 24 h
SP
RP
S
HP
Hydrolysis product (%)
Time (hrs)
B
5' AmpTTT 3'
3' Tp AAAGATCT(p) 5'
Flp(R308A)
C 0.5 1 2 3 6 8 16 24 h
Time (h)
C
SDS-PAGE
Urea-PAGE
Cre(R292K)
SP/RP mixture

Figure S2

Figure S2. Reactions of Arg-II mutants of Cre and Flp on MeP half-sites. The stereoisomers of MeP are compared to the phosphodiester in the schematic diagram at the top (as in Figure 1B). Note that O1 is intact in S_P-MeP, and so is O2 in R_P-MeP (see also Figure S1A, B). The representations of MeP-half-sites in this figure and subsequent ones are patterned after those in the main figures. The recombinase binding element is indicated by the parallel arrows, with the wavy lines at one end denoting extra nucleotides that are not relevant to the reaction. The position of MeP is indicated as 'mp'. The base pair abutting the scissile MeP is written in bold letters. This is the terminal base pair of the Flp binding element, and is the immediate neighbor of the Cre binding element. The 5' hydroxyl group of the bottom strand is phosphorylated (p). Reactions were performed on stereochemically pure forms or a racemic mixture of the indicated MeP half-sites, and product formation was followed with the help of the ^{32}P-label (asterisk) placed at the 5' end of the top strand. The hydrolysis product was analyzed (**A**-**C**) by phenol-chloroform extraction of the SDS-terminated reactions and ethanol precipitation of DNA, followed by electrophoresis in 12% denaturing (urea) polyacrylamide gels. As the phenol-chloroform extraction step would trap any cleaved covalent DNA-protein adduct at the interphase of the organic and aqueous layers, it was excluded from the analyzed DNA. The cleaved covalent intermediate was assayed (**C**) by 12% SDS-polyacrylamide electrophoresis of the terminated reactions. The bands corresponding to the unreacted substrate and the hydrolysis product are labeled as 'S' and 'HP', respectively. The cleaved covalent intermediate is drawn as a short line ending in a circle. The values in the kinetic plots are the Mean ± SD (standard deviation).

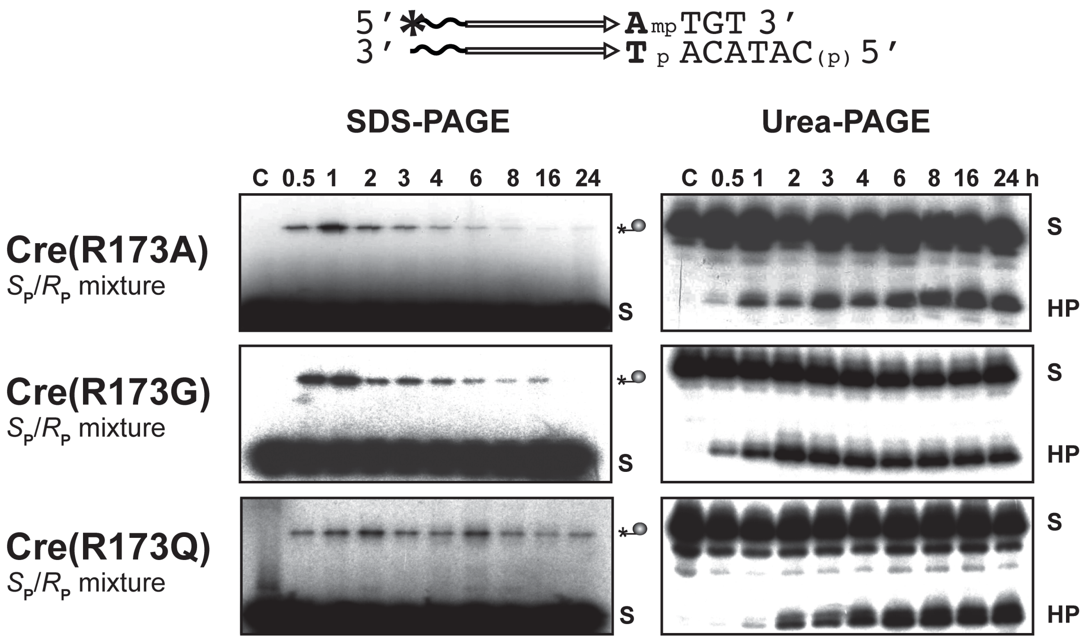

Figure S3

Figure S3. Reactions of Arg-I mutants of Cre on an MeP half-site. The reactions, performed on a racemic mixture of the MeP-half-site, were analyzed for the hydrolysis product or the cleaved covalent intermediate as described under Figure S2. In all the reactions with the Cre mutants (Arg-I as well as Arg-II mutants), the hydrolysis product was formed via the covalent intermediate.

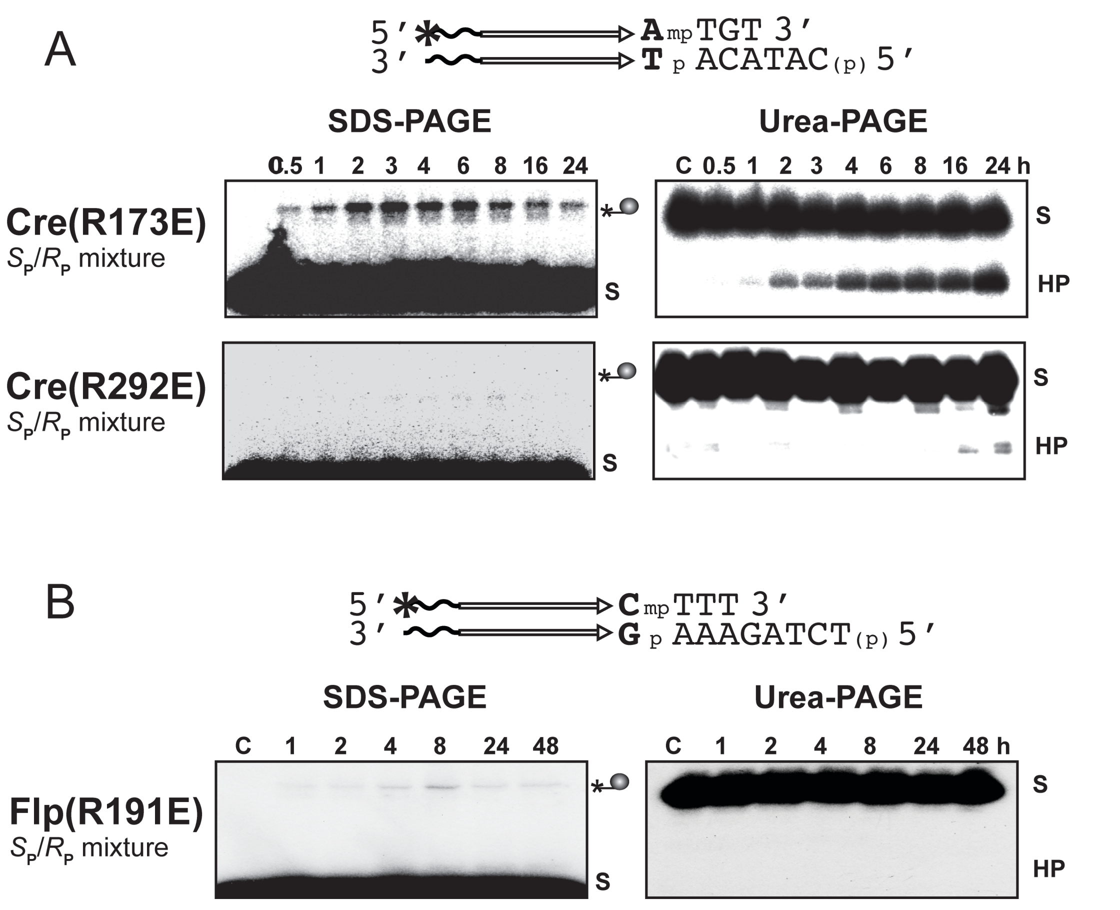

Figure S4

Figure S4. Effects of Arg-I or Arg-II to glutamic acid substitution in Cre and Flp on the MeP-half-site reaction. The indicated Cre mutants (**A**) and Flp(R191E) (**B**) were reacted with a racemic mixture of their respective MeP-half-sites. Formation of the cleaved tyrosyl intermediate and the hydrolysis product was analyzed by standard procedures (Figure S2, S3). Note that Cre(R173E) was active in yielding the covalent intermediate and converting it to the hydrolysis product. By contrast, Flp(R191E) and Cre(R292E) gave no detectable, or barely detectable, activity. As shown in Figure 6, Flp(R308E) exhibited strong hydrolytic activity. Thus, charge reversal at Arg-I or Arg-II has opposite effects on the functionality of Cre and Flp active sites.

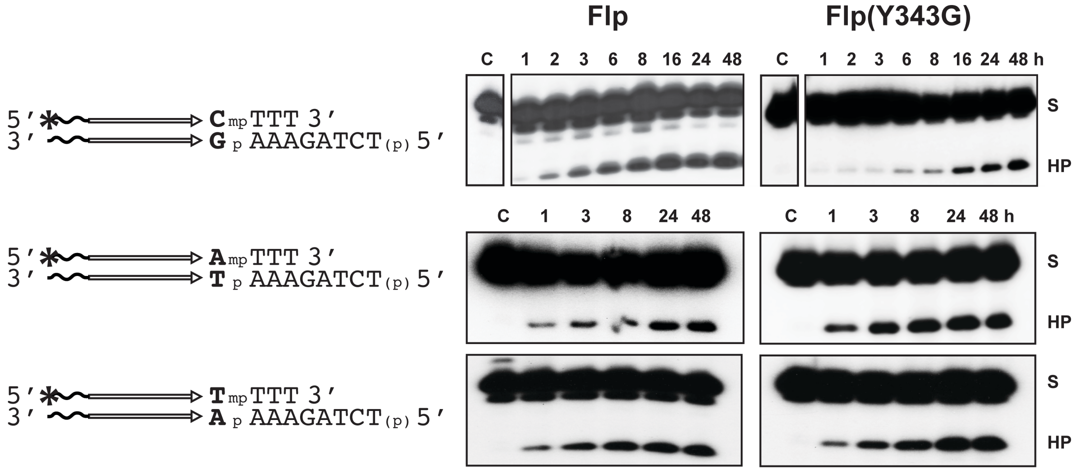

Figure S5

Figure S5. Reactions of Flp and Flp(Y343G) on half-sites containing C, A or T as the 5'-neighbor of MeP. The half-sites employed in these reactions differed only in the base 5'-adjacent to MeP and its complement on the non-scissile strand. The 5'-neighbor of the scissile phosphate in the native Flp target site is C. Substitution of this C by G (CG to GC transversion) leads to loss of activity in the phosphate containing site (1,2) and in the MeP-half-site (this study; data not shown). Each MeP-half-site was reacted with Flp and Flp(Y343G) under identical conditions. The product yield from Flp(Y343G) indicated the extent of direct hydrolysis. The product yield from the Flp reaction potentially included contributions from hydrolysis via the tyrosyl intermediate as well as direct hydrolysis. For the (CG)-MeP-half site, direct hydrolysis was a minor reaction, accounting for 25-30% of the total reaction. For both (AT)-MeP- and (TA)-MeP-half-sites, there was near equivalence in product amounts in the Flp and Flp(Y343G) reactions. Thus, direct hydrolysis was the only reaction, or the predominant one, with these substrates.

Oligonucleotide	Size (nt)	Sequence
**Cre**		
Scissile (top) strand	27	5' ACTTGGATCC**ATAACTTCGTATA**A(mp)tgt 3'
Non-scissile (bottom) strand	30	5' cataca**TTATACGAAGTTAT**GGATCCAAGT 3'
**Flp**		
Scissile (top) strand	26	5' ACTTGGATCC**GAAGTTCCTATAC**(mp)ttt 3'<br>5' ACTTGGATCC**GAAGTTCCTATAA**(mp)ttt 3'<br>5' ACTTGGATCC**GAAGTTCCTATAT**(mp)ttt 3'
Non-scissile (bottom) strand	31	5' tctagaaa**GTATAGGAACTTC**GGATCCAAGT 3'<br>5' tctagaaa**TTATAGGAACTTC**GGATCCAAGT 3'<br>5' tctagaaa**ATATAGGAACTTC**GGATCCAAGT 3'

Table S1. Synthetic oligonucleotides for the assembly of MeP-half-site substrates for Cre and Flp reactions. In the sequences of the oligonucleotides for the assembly of Cre half-sites, the bold letters denote the Cre binding element and the first base of the spacer sequence (abutting the strand exchange region). In the case of the Flp half-site oligonucleotides, the bold letters represent the Flp binding element. The letters in lower case (for both Cre and Flp half-site oligonucleotides) indicate the strand exchange region (in full in the non-scissile (bottom) strand; truncated in the scissile (top) strand). Sequences not directly relevant to the half-site reactions (drawn as wavy lines in the schematic representation of half-sites in figures) are shown in plain upper case letters.

REFERENCES

1. Senecoff, J.F., Rossmeissl, P.J. and Cox, M.M. (1988) DNA recognition by the FLP recombinase of the yeast 2 μm plasmid. A mutational analysis of the FLP binding site. *J Mol Biol*, **201**, 405-421.
2. Whiteson, K.L. and Rice, P.A. (2008) Binding and catalytic contributions to site recognition by Flp recombinase. *J Biol Chem*, **283**, 11414-11423.
